# Genetic regulation of lncRNA expression in whole human brain and their contribution to CNS disorders

**DOI:** 10.1093/bib/bbaf291

**Published:** 2025-06-21

**Authors:** Yijie He, Yaqin Tang, Pengcheng Tan, Dongyu Huang, Yongheng Wang, Tong Wen, Lin Huang, Jia Wang, Lizhen Shao, Qinyu Cai, Zhimou Li, Yueyang Wang, Taihang Liu, Zhijie Han

**Affiliations:** Department of Bioinformatics, School of Basic Medicine, Chongqing Medical University, 400016, Chongqing, China; Key Laboratory of Major Brain Disease and Aging Research (Ministry of Education), Chongqing Medical University, 400016, Chongqing, China; Department of Bioinformatics, School of Basic Medicine, Chongqing Medical University, 400016, Chongqing, China; Department of Bioinformatics, School of Basic Medicine, Chongqing Medical University, 400016, Chongqing, China; Department of Bioinformatics, School of Basic Medicine, Chongqing Medical University, 400016, Chongqing, China; Department of Bioinformatics, School of Basic Medicine, Chongqing Medical University, 400016, Chongqing, China; Department of Bioinformatics, School of Basic Medicine, Chongqing Medical University, 400016, Chongqing, China; Department of Bioinformatics, School of Basic Medicine, Chongqing Medical University, 400016, Chongqing, China; Department of Bioinformatics, School of Basic Medicine, Chongqing Medical University, 400016, Chongqing, China; Department of Bioinformatics, School of Basic Medicine, Chongqing Medical University, 400016, Chongqing, China; Key Laboratory of Major Brain Disease and Aging Research (Ministry of Education), Chongqing Medical University, 400016, Chongqing, China; Institute for Brain Science and Disease, Chongqing Medical University, 400016, Chongqing, China; Department of Bioinformatics, School of Basic Medicine, Chongqing Medical University, 400016, Chongqing, China; Institute for Brain Science and Disease, Chongqing Medical University, 400016, Chongqing, China; Department of Bioinformatics, School of Basic Medicine, Chongqing Medical University, 400016, Chongqing, China; Key Laboratory of Major Brain Disease and Aging Research (Ministry of Education), Chongqing Medical University, 400016, Chongqing, China; Department of Bioinformatics, School of Basic Medicine, Chongqing Medical University, 400016, Chongqing, China; Department of Bioinformatics, School of Basic Medicine, Chongqing Medical University, 400016, Chongqing, China; Key Laboratory of Major Brain Disease and Aging Research (Ministry of Education), Chongqing Medical University, 400016, Chongqing, China; Institute for Brain Science and Disease, Chongqing Medical University, 400016, Chongqing, China

**Keywords:** long non-coding RNAs, human brain, expression quantitative trait loci, functional analysis, central nervous system disorders

## Abstract

Long non-coding RNAs (lncRNAs) play a key role in the human brain, and genetic variants regulate their expression. Herein, expression quantitative trait loci of lncRNAs encompassing 10 brain regions from 134 individuals were analyzed, and novel variants influencing lncRNA expression (eSNPs) and respective affected lncRNAs (elncRNAs) were identified. The eSNPs showed proximity to their corresponding elncRNAs, enriched in the non-coding genome, and have a high minor allele frequency. The elncRNAs exhibit a high-level and complex pattern of expression. The genetic regulation is more tissue specific for lncRNAs than for protein-coding genes, with notable differences between cerebrum and cerebellum. Nonetheless, it shows relatively similar patterns across cortex regions. Furthermore, we observed a significant enrichment of eSNPs among variants associated with neurological disorders, especially insomnia, and identified insomnia-related lncRNAs involved in immune response functions. Moreover, the present study offers an improved tool for lncRNA quantification, a novel approach for lncRNA function analysis, and a database of lncRNA expression regulation in human brain. These findings and resources will advance the research on non-coding gene expression regulation in neuroscience.

## Introduction

Long non-coding RNAs (lncRNAs) constitute a class of non–protein-coding RNAs with a length of >200 nucleotides. These lncRNAs are highly represented in the human genome and widely involved in various key cellular and biological processes [1]. Previous studies have shown that the expression of lncRNAs has greater intra-individual heterogeneity and higher tissue specificity than protein-coding genes [2, 3]. Importantly, lncRNAs also exhibit significantly high transcript abundance in the brain compared to the other human tissues and critically regulate the development of the human brain, neuronal regeneration, maintenance of synaptic plasticity, and pathogenesis of central nervous system (CNS) disorders [4–8]. Strikingly, >40% of currently known lncRNAs are specifically expressed in different regions of the human brain, according to statistics from the GENCODE (v39) [9]. LncRNADisease (v2) predicted that ~90% of the total lncRNAs are associated with at least one CNS disorder, although <2% of these have been rigorously confirmed by molecular biology experiments [10].

Moreover, millions of common single nucleotide polymorphisms (SNPs) exist in the human genome. These SNPs are major factors contributing to individual differences in behavioral traits and disease susceptibility. The genome-wide association studies (GWASs) have identified a large number of SNPs significantly associated with brain development and CNS disorders [11]. They exhibit marked tissue specificity [12] and are mainly located in the non-coding regions of the genome (approximately 93%), especially the lncRNA sequences [13–15]. Importantly, most of these non-coding SNPs play key roles in the regulation of lncRNA expression, which is a prerequisite for acting on proximal protein-coding target genes indirectly, further contributing to the pathogenesis of many CNS disorders [16–18]. For example, the A allele of rs1950834 serves as a protective variant against major depressive disorder (MDD). A key feature of the mechanism is that the protective allele reduces chromatin accessibility by disrupting the binding of transcription factor ETV1, consequently decreasing the expression of lncRNA AL121821.1 in neural progenitor cells [19, 20]. The minor alleles of three GWAS-identified risk variants (rs7704909, rs12518194, and rs4307059 localized in the gene-poor 5p14.1 region) are significantly associated with autism spectrum disorder (ASD), synergistically promoting the expression of lncRNA MSNP1AS. This lncRNA is highly enriched in the postmortem cerebral cortex of ASD patients, inhibiting the production of moesin and resulting in the dysregulation of neuronal architecture [21].

Based on the above findings, we can infer that the regulation of lncRNA expression by genetic variation in the brain is crucial for the vital neuronal functions and the pathogenesis of CNS disorders. The expression quantitative trait loci (eQTL) analysis has been used extensively as a robust multi-omics integration tool for systematic analysis of the genome-wide correlations between altered gene expression and genetic variations in a specific tissue. Previous studies have applied eQTL analysis to investigate the regulation of gene expression in the human brain. However, the studies mainly focused on the protein-coding genes or involved only the single brain tissues. For example, xQTL Serve [22], BrainCloud [23], and Myers *et al.* [24] explored the effect of genetic variants on the expression of protein-coding genes in the prefrontal cortex. Although the samples used in SNCID [25], BRAINEAC [26], GTEx [27], and MetaBrain [28] include multiple brain tissues, the former two are concerned only with the expression and regulation of protein-coding genes, and the latter two lack targeted investigations on the functional characterization of eQTL lncRNAs and their related CNS disorders.

**Figure 1:**
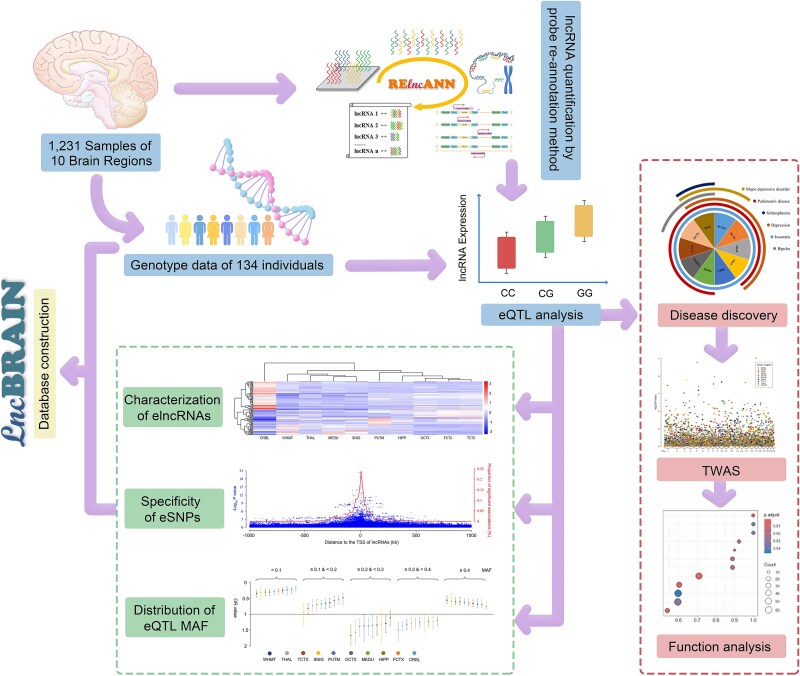
A flowchart of the study to profile the regulation of lncRNA expression in the human brain. The microarray probe re-annotation method was improved to quantify lncRNAs across 10 distinct brain regions, utilizing a dataset comprising 1231 samples sourced from UKBEC. Then, a *cis*-eQTL analysis of the lncRNAs was performed using the quality-controlled genotype data from the same individuals. Subsequently, the regulation of lncRNA expression across diverse brain regions was comprehensively characterized from multiple perspectives. Finally, a novel approach was designed to identify potential CNS disorder–related lncRNAs and determine their functions, leveraging eQTL results. Moreover, the findings and analytical models of this study were stored in a user-friendly visual database lncBRAIN and the probe re-annotation tool was integrated into a web server RElncANN for future researchers use in neuroscience research.

In the present study, we addressed these issues using large-scale multi-omics data from the UK Brain Expression Consortium (UKBEC), which comprises 10 brain regions of European-descent individuals without neurological disorders [26]. First, lncRNA identification and quantification were conducted using an improved microarray probe re-annotation method. Next, we conducted a rigorous genome-wide eQTL analysis by integrating the quantified lncRNAs with quality-controlled genotype data. We also characterized and compared the profiles of the regulation of genetic variants on lncRNA expression across brain regions. Finally, a new approach and trained prediction models based on the eQTL results were developed to identify the potential CNS disorder–related lncRNAs and delineate their functions (Fig. 1). In addition, we constructed user-friendly web servers to store the lncRNA eQTL results, trained prediction models, and analysis tools.

## Results

### LncRNA identification and quantification

First, we obtained raw CEL files from 1231 samples derived from 134 donors of European descent without neurological disorders, involving 10 brain regions: frontal cortex (FCTX), occipital cortex (OCTX), temporal cortex (TCTX), cerebellar cortex (CRBL), hippocampus (HIPP), medulla inferior olivary nucleus (MEDU), substantia nigra (SNIG), putamen at anterior commissure (PUTM), thalamus at lateral geniculate nucleus (THAL), and intralobular white matter (WHMT). The statistics of the samples are summarized in Supplementary Methods and Table S1.

Then, we generated the product quality control metrics of the array. The signal intensity of the quality control probes shows the expected rank order for all 1231 samples, i.e. BioB < BioC < BioD < Cre (Supplementary Fig. S1). Next, we identified and quantified 11 587 lncRNAs (~74.2% of all lncRNAs according to the annotation of Ensembl hg38 release 82) from the1231 exon arrays using an improved probe set re-annotation strategy (see Supplementary Methods for details) [29–31]. These lncRNAs consisted of 6222 lincRNAs (53.70%), 4141 antisense lncRNAs (35.74%), 693 sense intronic lncRNAs (5.89%), 376 processed lncRNAs (3.25%), 154 sense overlapping lncRNA (1.33%), and a macro lncRNA (unspliced lncRNAs that are several kb in size) (5.89%) (Supplementary Table S2). The proportions of the six identified lncRNA subtypes by re-annotation were consistent with their proportion in the total lncRNAs (50.95%, 36.64%, 6.03%, 5.12%, 1.25%, and 0.01%, respectively) (Supplementary Fig. S2a and b). The overall expression level of these identified lncRNAs was similar across the 10 brain regions (Supplementary Fig. S3). Finally, we evaluate the quality of the improved probe set re-annotation approach. The probe coverage of this study was ~16.33 probes per lncRNA. Compared to previous re-annotation methods, the lncRNA recognition rate and average probe coverage are ~10.28% and 13.23 [32], 64.37% and 19.83 [33], and 86.94% and 3.35 [34], respectively. According to the results, our method outperformed most of the existing methods in each aspect. Additionally, Zheng *et al.*’s method allows for one-base mismatch during the sequence alignment process [34]. Therefore, our improved probe set re-annotation approach is advantageous in lncRNA discovery and quantification (Supplementary Fig. S2c). We also provided an online probe re-annotation tool RElncANN for identifying and quantifying human lncRNAs from the multi-platform microarray data (https://relncann.cqmu.edu.cn/appcel/).

### Identification and validation of *cis*-eQTLs in lncRNAs across brain regions

Through *cis*-eQTL analysis, 440 lncRNAs were identified; their expressions were significantly affected by *cis*-eQTLs in at least one brain region (elncRNAs). The number of elncRNAs was from a minimum of 70 (SNIG) to a maximum of 172 (CRBL) in the 10 brain regions (Supplementary Table S3). Finally, we used the first 5% of the permuted minimum *P* values as a threshold to discover the variants with significant regulatory effects (eSNPs) in each lncRNA, as described previously [27, 35]. A total of 25 645 eSNPs were discovered in at least one brain region (from a minimum of 2367 in SNIG to a maximum of 9264 in CRBL) (Supplementary Table S4). Moreover, a user-friendly lncBRAIN database was constructed to visualize the lncRNA expression, eQTL results, and linkage disequilibrium (LD) of the corresponding variants in the 10 brain regions (http://lncbrain.org.cn/) [36].

**Figure 2:**
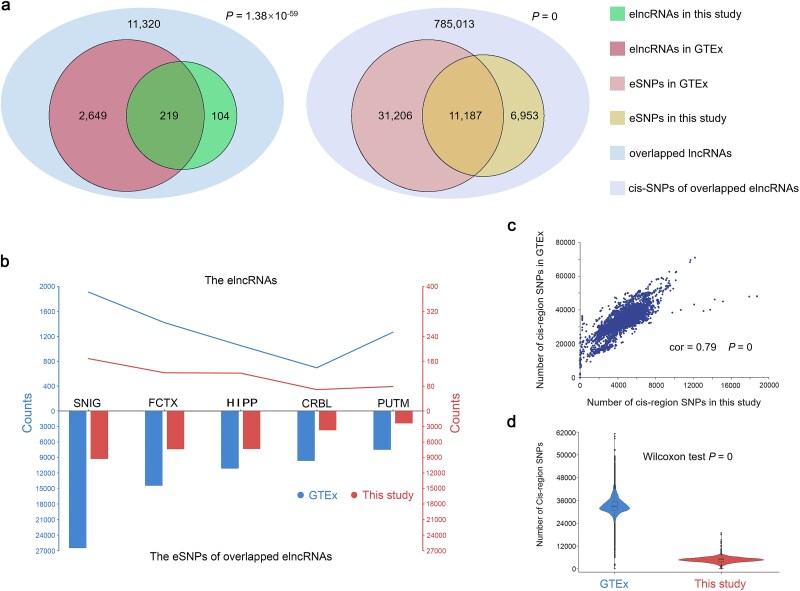
Comparison of the *cis*-eQTL signals between GTEx and this study. (a) The replication of the total elncRNAs (left) and eSNPs of overlapped elncRNAs (right) in the five brain regions (i.e. CRBL, FCTX, HIPP, SNIG, and PUTM) identified by this study in that of GTEx (v8), respectively. The statistical significance was determined by a hypergeometric distribution test with the 11 320 common lncRNAs and the 785 013 *cis*-SNPs of overlapped elncRNAs as the background, respectively. (b) The number of elncRNAs and eSNPs in overlapped elncRNAs in each brain region identified by this study and GTEx, respectively. The left *y*-axis represents GTEx, while the right *y*-axis corresponds to this study. (c) The quantitative correlation of the *cis*-SNPs between this study and GTEx. Each dot represents the number of SNPs in the *cis* region of an lncRNA. (d) The violin plots show the quantitative distribution of *cis*-SNPs per lncRNA in the two studies.

In order to confirm the repeatability of our findings, we evaluated the consistency of the identified elncRNAs and corresponding eSNPs with GTEx datasets (v8) among the five shared brain regions, i.e. CRBL, FCTX, HIPP, SNIG, and PUTM. The elncRNAs and eSNPs were collectively identified by integrating the results from the five brain regions. For elncRNAs, we selected 11 320 lncRNAs common to this study and GTEx as the background. Figure 2a demonstrates a significant overlap of elncRNAs identified in this study with those from GTEx, as indicated by the hypergeometric distribution test (*P* = 1.38 × 10^−59^). Notably, approximately one-third of the elncRNAs are newly discovered. We also observed similar results in the five brain regions, respectively (Supplementary Fig. S4). Subsequently, our comparative expression analysis in the overlapped region demonstrated that the novel elncRNAs we identified exhibited significantly lower expression levels compared to GTEx’s eGenes (Supplementary Fig. S5). For eSNPs, the SNPs located in *cis* regions of the overlapped elncRNAs were selected as background. A significant overlap of eSNPs was observed between the two datasets (hypergeometric distribution test *P =* 0) and nearly 40% of our identified eSNPs were novel (Fig. 2a), which is also consistent with the results of the five brain regions (Supplementary Fig. S6). In addition, we found that both the recognition rates of elncRNAs and the corresponding eSNPs in different brain regions showed the same trend in our study and that of GTEx (cor = 0.84 and 0.83, *P* = 3.64 × 10^−2^ and 4.16 × 10^−2^, respectively) (Fig. 2b). Next, we compared the number and distribution of the *cis*-SNPs used for eQTL analysis in this study and GTEx and found that the number of *cis*-SNPs of the 11 320 common lncRNAs in the two studies have a strong linear correlation (cor = 0.79, *P* = 0) (Fig. 2c). Nonetheless, the total number of *cis*-SNPs used in this study is significantly less than that of GTEx (two-tailed Wilcoxon test *P* = 0) (Fig. 2d), which might be the primary cause of fewer elncRNAs and eSNPs identified in this study compared with GTEx. In summary, the results above reveal a significant replication and general reliability of our findings.

### Characterization and expression patterns of elncRNAs in different brain regions

**Figure 3:**
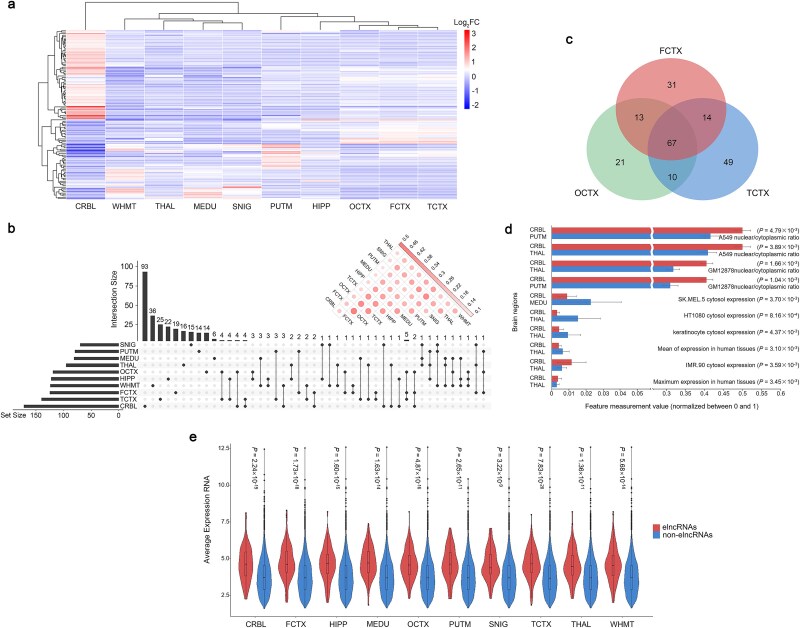
Characteristics of elncRNAs in the human brain. (a) Heatmap shows the differential expression pattern of elncRNAs among the 10 brain regions. (b) UpSet plot shows the number of elncRNAs obtained from different brain regions and their tissue specificity and intersection. Corrplot in the upper right corner reveals the Jaccard index of elncRNAs among the 10 brain regions. The large circles with dark shades indicate high similarity. (c) Venn diagram shows the overlap of elncRNAs identified in the cerebral cortex regions, i.e. OCTX, FCTX, and TCTX, respectively. (d) The top 10 most significant differences in characteristic features between the elncRNAs from distinct brain regions. Each bar represents the average measurement of a feature in a specific brain region. The overall results are summarized in Supplementary Table S8. (e) Violin plots show the average expression of each elncRNA and non-elncRNA across individuals in the 10 brain regions.

We analyzed the expression profiles and features of the 440 elncRNAs across the 10 brain regions. A differential expression analysis revealed that the proportion of differentially expressed lncRNAs in the 440 elncRNAs was significantly higher than that in the total 11 587 lncRNAs quantified (odds ratio (OR) = 3.36, *P* = 2.20 × 10^−16^); the results were consistent across the 10 brain regions (Supplementary Table S5). In addition, elncRNAs showed a marked difference in expression between CRBL and the remaining brain regions, while it was mostly similar among the three cerebral cortex regions (i.e. OCTX, FCTX, and TCTX) (Fig. 3a). Then, we compared the distributions of the 440 elncRNAs and assessed their similarity among the 10 brain regions using the Jaccard index. Figure 3b shows minor differences in elncRNA counts across the 10 brain regions, with CRBL having the highest abundance. Notably, the number of elncRNAs in CRBL is substantially higher than in the other regions. The Jaccard index indicated that the similarity is the lowest between CRBL and the other nine brain regions in terms of elncRNAs. It also reveals the highest overall similarity among the three cerebral cortex regions, which is consistent with the findings of the differential expression analysis. Specifically, 119, 125, and 140 elncRNAs were identified in OCTX, FCTX, and TCTX, respectively. Approximately 31.46% of elncRNAs were shared among the three regions, while 48.83% were found in at least two regions (Fig. 3c). Next, we employed a two-tailed Wilcoxon test to assess the differences in characteristic features between the elncRNAs from distinct brain regions; 76 lncRNA features were obtained from the LnCompare database. The results indicated persistent differences in the features, mainly between the CRBL and other brain regions. A total of 39 lncRNA features differed significantly between at least one pair of brain regions (*P* < .05), and ~90% of these were involved in CRBL. These feature differences were associated with gene/exon length, cell regulation, potential risk variant counts, and distribution across tissues and cell lines (Fig. 3d and Supplementary Table S8). Finally, we compared the average expression and fluctuations in expression between elncRNAs and non-elncRNAs (empirical *P* > .05) in each brain region using the two-tailed Wilcoxon test, respectively. The elncRNAs consistently showed a significantly higher average expression level and variance across individuals than the non-elncRNAs in all 10 brain regions (*P* < .05) (Fig. 3e and Supplementary Fig. S7). This finding was further substantiated by calculating the expression standardized residuals (*Z*-scores) of the lncRNAs used for eQTL analysis to evaluate their expression levels and fluctuations comprehensively. The *Z*-scores of each lncRNA in each sample were calculated by regressing the gender, first 3 principal components, and first 15 probabilistic estimation of expression residuals (PEERs) from lncRNA expression levels. The lncRNAs with absolute median *Z*-score across brain regions greater than two in at least one individual were defined as the outlier (see Supplementary Methods for details) [37]; 322 outliers were detected (Supplementary Table S9). The results of the hypergeometric distribution test showed significant overlap (*P* < .05) between the elncRNAs and the outliers in each brain region, respectively (Supplementary Fig. S8). Taken together, the effect of genetic variation on lncRNA expression is specific in the cerebellum and similar between different regions of the cerebral cortex. Interestingly, the lncRNAs with high expression and tissue specificity in brain are likely elncRNAs.

### Tissue-specific genetic regulation of lncRNA expression across brain regions

**Figure 4:**
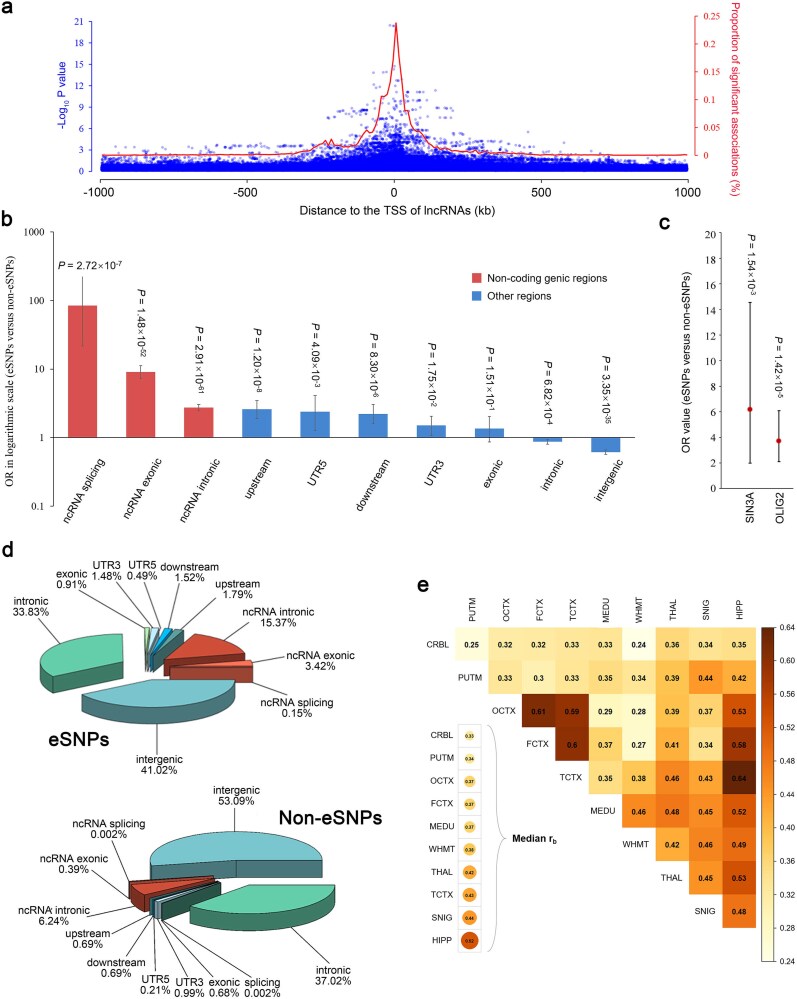
Specificity of genetic regulation on lncRNA expression in the human brain. (a) Significance of eQTLs and percentage of eSNPs with respect to the distance from variants to TSS of corresponding lncRNAs in the whole brain. Dots represent the variants in cis regions with the negative log-transformed *P* values of eQTL analysis (left *y*-axis). The solid line represents the percentage of eSNPs per 10-kb window (right *y*-axis). The results of each brain region are displayed in Supplementary Fig. S10. (b) Enrichment analysis of the eSNPs among 10 functional categories of variants (i.e. ncRNA splicing, ncRNA intronic, ncRNA exonic, upstream, downstream, 5′-UTR, 3′-UTR, exonic, intronic, and intergenic variants) in the whole brain measured by the two-tailed Fisher’s exact test. The variants in non-coding genomic regions were marked red, while the others were marked blue. The black bars in the histogram indicate 95% CI. The results of each brain region are displayed in Supplementary Fig. S11. (c) Enrichment analysis of the eSNPs among the ChIP-seq peaks of two TFs (i.e. SIN3A and OLIG2) in FCTX by two-tailed Fisher’s exact test similar to subfigure b. (d) Pie charts indicate the percentage of each functional type of variants in the datasets of eSNPs and non-eSNPs, respectively. The variants in non-coding genomic regions are shown in warm colors, while the others are shown in cool colors. (e) Estimated correlation of genetic regulation on lncRNA expression between two of the 10 brain regions. Heatmap of the cells shows the correlation of beta values (${r}_b$). The median of the ${r}_b$ between each brain region and the remaining nine areas is displayed in the bottom left corner.

The specificity of genetic regulation on lncRNA expression in different brain regions was assessed by eSNP profiling. First, consistent with the tissue specificity of the elncRNAs, the eSNPs unique to CRBL significantly exceeded those in other brain regions (a similar number of eSNPs in the 10 brain regions) (Supplementary Fig. S9). Then, we explored the association between the effect of variant on lncRNA expression and its distance from the transcriptional start site (TSS) of the corresponding lncRNA. The results showed that the eSNPs tend to be clustered around the TSS of lncRNA, especially for the variants with lower *P* values in the *cis* regions (Fig. 4a). These findings are consistent across the 10 brain regions (Supplementary Fig. S10). Next, the genotyped variants were annotated using ANNOVAR software and underwent LD-based filtering with PLINK. The independent variants in *cis* regions with the eQTL nominal *P* > .5 were selected and defined as the non-eQTL SNPs (see Supplementary Methods for details). A total of 2628 eSNPs and 1 927 132 non-eSNPs were uniquely annotated as one of the 11 functional class and independent of each other in whole brain. The results showed that the percentage of variants located in non-coding genomic regions (i.e. ncRNA intronic, ncRNA exonic, and ncRNA splicing) is significantly higher in the eSNP set (15.37%, 3.42%, and 0.15%, respectively) compared to the non-eSNP set (6.24%, 0.39%, and 0.002%, respectively) (Fig. 4d). By comparing the proportions of each functional class between eSNPs and non-eSNPs using the two-tailed Fisher’s exact test with a threshold of *P* < .05, we found that the eSNPs are significantly and most highly enriched among variants in ncRNA splicing (OR = 84.00, *P* = 2.72 × 10^−7^), ncRNA exonic (OR = 9.03, *P* = 1.48 × 10^−52^), and ncRNA intronic regions (OR = 2.73, *P* = 2.91 × 10^−61^) (Fig. 4b). Similar results were observed in each of the 10 brain regions (Supplementary Fig. S11). Furthermore, to assess whether the eSNPs of lncRNAs are enriched in the regulatory elements (e.g. promoter and enhancer) mainly located in the non-coding genomic sequences, ChIP-seq peak data for human transcription factor (TF) binding sites in FCTX from ReMAP (dataset “brain-prefrontal-cortex 2022,” containing two TFs: SIN3A and OLIG2 [38]) were used for the two-tailed Fisher’s exact test (see Supplementary Methods for details). The results showed a significant positive enrichment of the eSNPs in both TF SIN3A (OR = 6.17, *P* = 1.54 × 10^−3^) and OLIG2 binding site (OR = 3.71, *P* = 1.42 × 10^−5^) compared with the non-eSNPs (Fig. 4c). Similar to the results of the previous step, the regulation of lncRNAs by genomic variants mainly involves non-coding functional sequences, which is different from that for protein-coding genes [39]. Finally, we investigated the tissue specificity of genetic regulation on lncRNA expression in different brain regions. The top-associated eSNPs for each of the elncRNAs in the 10 brain tissues were selected to compare the similarity of the genetic effects (measured by beta) between the two tissues, respectively, after correcting for the estimated errors of eQTL analysis (see Supplementary Methods for details). Strikingly, a maximal difference was observed in the genetic effects on lncRNA expression between CRBL and the remaining brain regions (median ${r}_b$ = 0.33), while it was similar among the three cerebral cortex regions (i.e. OCTX versus FCTX: ${r}_b$ = 0.61, TCTX versus FCTX: ${r}_b$ = 0.60, OCTX versus FCTX: ${r}_b$ = 0.59) (Fig. 4e). The correlation of genetic effects among different brain regions is consistent with the characteristic of elncRNAs. Moreover, the genetic regulation on lncRNA expression is relatively weakly correlated among brain regions compared to coding genes [40], indicating a more pronounced tissue specificity of the former. In summary, variants in the proximity of TSS and located in the non-coding genomic regions might affect the expression of lncRNAs in the human brain through tissue-specific regulation.

### Association between variant frequency and lncRNA *cis*-eQTL regulation

**Figure 5:**
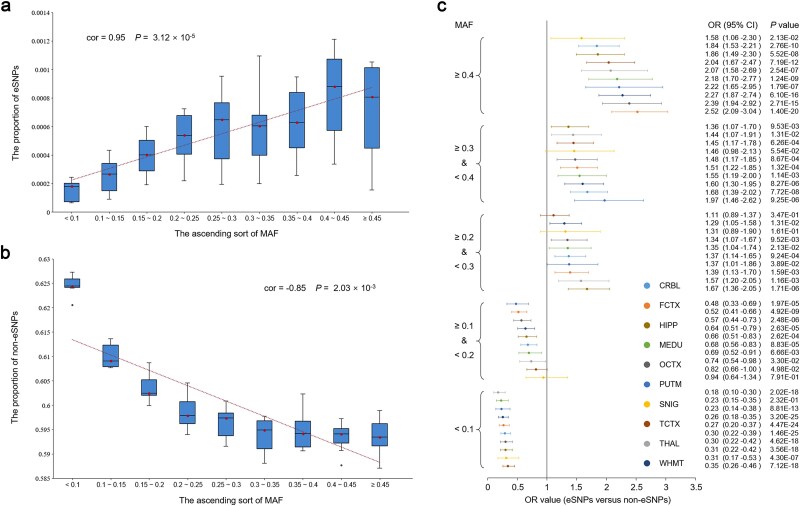
Correlation between variant frequency and their regulatory effect on lncRNA expression in the human brain. (a) and (b) reveal an increasing and decreasing proportion of eSNPs and non-eSNPs among *cis*-SNPs with increasing MAF, respectively. The box plots show the distribution of these proportions among the 10 brain regions in the corresponding MAF bin. The red dotted lines are the linear regression results for the median of the proportions. (c) The forest plot shows enrichment results of eSNPs in each different MAF bin by comparing them with the non-eSNPs. The OR and 95% CI were calculated by a two-tailed Fisher’s exact test. Different colors represent different brain regions.

We analyzed the distribution of LD-pruned eSNPs and non-eSNPs across different minor allele frequency (MAF) bins to investigate the relationship between variant frequency and their regulatory effect on lncRNA expression in the human brain. For the whole brain, the median of the proportion of eSNPs increases significantly with increasing MAF (cor = 0.95, *P* = 3.12 × 10^−5^) (Fig. 5a), whereas a significant negative correlation was established between the median of the proportion of non-eSNPs and their MAF (cor = −0.85, *P* = 2.03 × 10^−3^) (Fig. 5b). The consistent results were observed in the 10 brain regions (Supplementary Figs. S12 and S13). Subsequently, we performed a two-tailed Fisher’s exact test to compare the proportion of the LD-pruned eSNPs and non-eSNPs in each MAF bin (see Supplementary Methods for details). Interestingly, the eSNPs were enriched in variants with higher MAF, while the non-eSNPs are more likely to be enriched in variants with lower MAF in the 10 brain regions (Fig. 5c and Supplementary Fig. S14). Taken together, the altered lncRNA expression profiles in the human brain could be attributed to the regulatory effects of common variants.

### Disease association of the *cis*-eQTLs by risk loci enrichment

**Figure 6:**
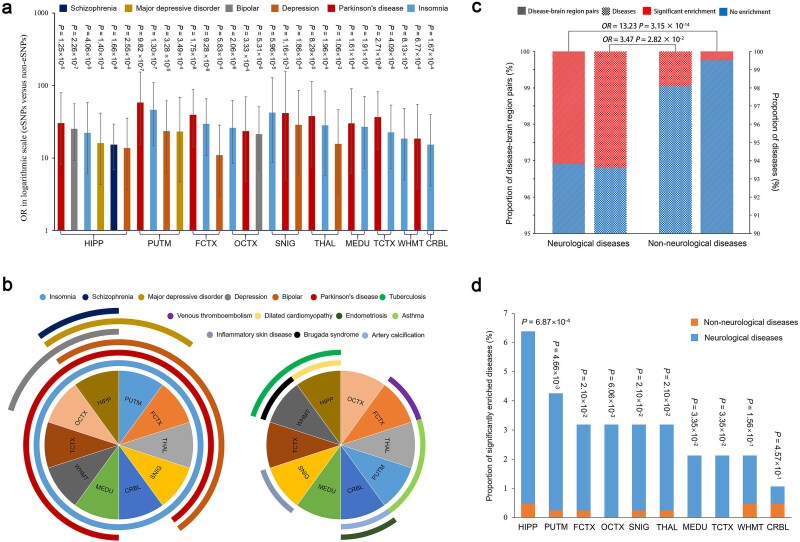
Enrichment of eSNPs among neurological and non-neurological disease–related loci in 10 brain regions. (a) The 29 significant enrichment results of neurological diseases (i.e. insomnia, MDD, PD, schizophrenia, depression, and bipolar) in the 10 brain regions. The two-tailed Fisher’s exact test was employed to explore the enrichment of the eSNPs among 511 diseases (average ~1281 associated SNPs per disease) compared with non-eSNPs. (b) A significantly higher identification rate of SNP-enriched neurological diseases compared with non-neurological diseases. Two-tailed Fisher’s exact test was used to compare the proportion of SNP-enriched neurological and non-neurological diseases, and SNP-enriched neurological disease–brain region pairs and non-neurological disease–brain region pairs in their respective groups. (c) The distribution of SNP-enriched neurological and non-neurological diseases in the 10 brain regions, respectively. Curve coverage means a significant enrichment of eSNPs among the corresponding disease-related loci in specific brain regions. (d) The identification rate of SNP-enriched neurological and non-neurological diseases in the 10 brain regions. Two-tailed Fisher’s exact test was used to assess their difference.

The basic concept of GWAS is that the common variants tend to contribute to the pathogenesis of common and complex diseases [41]. Given the results of our above analysis and the tissue specificity of eQTL and disease, we speculate that the regulation of lncRNA expression by genetic variability in the brain could be associated with neurological diseases. Thus, we conducted a risk loci enrichment analysis in various human diseases using the association hits (*P* < 1 × 10^−5^) from GWAS Catalog to assess the potential contribution of the non-coding genetic regulation to disease risks and compare the specificity between neurological and non-neurological diseases. The risk loci enrichment analysis revealed that the regulation of lncRNA expression by genetic variability in the brain is strongly associated with neurological diseases. Furthermore, 29 significantly SNP-enriched neurological disease–brain region pairs including six neurological diseases (~6.4% of all neurological diseases) were identified: insomnia (mean OR = 27.73), MDD (mean OR = 19.49), Parkinson’s disease (PD) (mean OR = 35.07), schizophrenia (mean OR = 15.21), depression (mean OR = 18.45), and bipolar (mean OR = 23.27) (Fig. 6a). Conversely, only 10 such non-neurological disease–brain region pairs (involving ~1.9% non-neurological diseases) showed a close correlation between the lncRNA eSNPs identified in brain tissues and neurological diseases with respect to the diseases (OR = 3.47, *P* = 2.82 × 10^−2^) and the disease–brain region pairs (OR = 13.23, *P* = 3.15 × 10^−14^) (Fig. 6b). Figure 6c shows that the SNP-enriched neurological diseases encompass more brain regions than the non-neurological diseases. For example, insomnia- and PD-related SNPs were significantly enriched in 10 and 9 brain regions, respectively, whereas most non-neurological diseases typically involved only one brain region. Notably, HIPP eQTLs contributed to all six SNP-enriched neurological diseases, whereas CRBL associations were specific to insomnia. Finally, Fig. 6d illustrates a higher proportion of significantly SNP-enriched diseases within the category of neurological diseases compared with the non-neurological diseases across most of the 10 brain regions, and most significant difference appeared in the HIPP (*P* = 6.87 × 10^−4^). In summary, these results showed that the influence of genomic variants on lncRNAs might contribute to disease pathogenesis and is mainly associated with neurological diseases; this phenomenon is consistent with the utilization of brain tissue samples in this study.

### Identification and functional implications of insomnia-related lncRNAs

**Figure 7:**
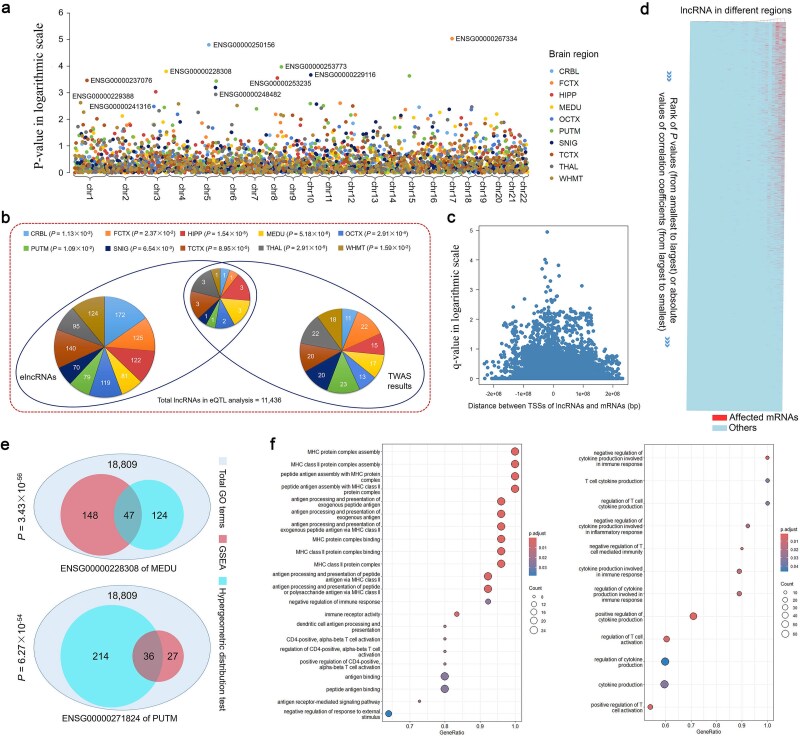
The exploration of insomnia-related lncRNAs and their functions based on eQTL data. (a) the Manhattan plot displays the insomnia TWAS outcomes for lncRNAs across 10 distinct brain regions. The identified lncRNAs with the smallest *P* value in each brain region are labeled in the figure. (b) The significant consistency between elncRNAs and TWAS results in each brain region was assessed using the hypergeometric distribution test. The distinct brain regions are indicated by different colors. (c) The significance of the effect of the lncRNA on mRNA expression is associated with the distance between its TSS when they are located on the same chromosome. (d) The heatmap shows the distribution of the mRNAs whose expression is significantly affected by the lncRNAs identified by 2SLS analysis. The *x*-axis represents the descending order of the absolute values of the correlation coefficients calculated by co-expression analysis between lncRNAs and mRNAs (or ascending order of *P* values). The affected mRNAs are mainly concentrated in high-correlation areas. (e) The significant overlap between the lncRNA-related pathways identified by GEAS and hypergeometric algorithm was evaluated using the hypergeometric distribution test. (f) The dot plots depict the immune-related pathways (i.e. regulation of cytokine production, T cell–mediated immunity, and antigen processing and presentation) in which two lncRNAs significantly associated with insomnia are implicated.

Since only insomnia exhibited significant involvement across all 10 brain regions in the risk loci enrichment analysis (Fig. 6c), we chose insomnia as a representative example to discover the disease-related lncRNAs and investigate their functions based on the eQTL data. First, we utilized eQTL data to train the transcriptome-wide association studies (TWASs) prediction model of lncRNAs using PredictDB, and then combined it with a large-scale GWAS dataset (*n* = 1 331 010) to identify insomnia-related lncRNAs in the 10 brain regions. Subsequently, 169 lncRNAs potentially related to insomnia (*P* < .05), including eight with FDR *q* < 0.1 (Benjamini–Hochberg adjustment), were identified (Fig. 7a and Supplementary Table S11). These lncRNAs are implicated in all 10 brain regions and do not exhibit any chromosome specificity; however, they are consistent with the elncRNAs in each brain region based on the hypergeometric distribution test (*P* < .05) (Fig. 7b), indicating a critical role of genetic regulation on lncRNA expression in disease mechanisms.

Since lncRNAs exert their functions by regulating mRNA expression, we designed an approach based on two-stage least squares (2SLS) estimation to discern this causal influence. The 16 945 protein-coding genes were quantified in the same samples used for lncRNA quantification (see Supplementary Methods for details). In nine brain regions (excluding OCTX), 25 out of the 169 insomnia-associated lncRNAs significantly regulated ≥1 mRNA, with a median of ~22 affected mRNAs per lncRNA (the threshold of FDR *q* < 0.05, Benjamini–Hochberg adjustment) (Supplementary Table S12). A proximal localization (measured by the distance between their TSSs) of the lncRNA and mRNA on the same chromosome would further enhance the influence effect (Fig. 7c). Moreover, a co-expression analysis was conducted to calculate the Pearson correlation coefficient of expression between the 25 lncRNAs and 16 945 protein-coding genes pairwise in the corresponding brain regions (Supplementary Table S13). Strikingly, the results of 2SLS estimation were consistent with those of co-expression analysis. The majority of the affected mRNAs identified through 2SLS estimation exhibit a pronounced tendency to display stronger co-expression with their corresponding lncRNAs (higher absolute value of Pearson correlation coefficient and smaller *P* values), underscoring the reliability of the 2SLS estimations (Fig. 7d).

Finally, based on the affected mRNA, we employed two enrichment methods, gene set enrichment analysis and hypergeometric distribution test, to deduce the functions of insomnia-related lncRNAs. The Gene Ontology (GO) dataset was used as the reference, and two lncRNAs, ENSG00000228308 (in MEDU) and ENSG00000271824 (in PUTM), were selected according to the following criteria: TWAS *q* < 0.1 and number of affected mRNAs > 20. Interestingly, the enriched terms are mainly involved in immune-related pathways, including regulation of cytokine production, T cell–mediated immunity, and antigen processing and presentation (FDR threshold *q* < 0.05, Benjamini–Hochberg adjustment) (Fig. 7e and Supplementary Tables S14 to S15). Previous studies demonstrated that the human brain contains immune signaling molecules that interact with the neurochemical systems and regulate normal sleep patterns [42]; e.g. cytokines have been widely known to play a crucial role in regulating sleep–wake behavior [42, 43]. Several genetic variants associated with sleep duration have been identified within the genes involved in cytokine signaling [44], and antagonizing cytokines (such as interleukin-1 (IL-1) and tumor necrosis factor (TNF) in human CNS, as well as upd2 in *Drosophila*) can increase the risk of insomnia through endocrine and inflammatory regulation [42, 45]. On the other hand, a negative feedback mechanism was discovered wherein sleep deprivation triggers the efflux of prostaglandin D2 across the blood–brain barrier, leading to a cytokine storm–like syndrome and severe inflammation in the peripheral immune system [46, 47]. Some studies also noted the interaction between the CD4^+^ T cells and sleep behavior disorders [48, 49]. Therefore, dysregulation of these lncRNAs could be a potential contributing factor to the aforementioned mechanisms of insomnia.

## Discussion

LncRNAs are prevalent in the human genome and intricately involved in diverse cellular and biological processes [1]. Unlike protein-coding genes, lncRNAs exhibit greater intra-individual heterogeneity and tissue specificity [2, 3]. Particularly enriched in the human brain, lncRNAs are key regulators in brain development, neuronal regeneration, synaptic plasticity maintenance, and CNS disorder pathogenesis [4–8]. Many of the variants associated with brain-related traits identified by GWAS are localized in the non-coding genome and regulate lncRNA expression [11–18]. Understanding the genetic regulation of lncRNAs in the brain is crucial for deciphering neuronal functions and CNS disorder mechanisms. Most eQTL analyses focus on protein-coding genes or single brain tissues, lacking in-depth investigations into the functional characterization of eQTL lncRNAs and their contributions to CNS disorders across the brain [22–28].

In the present study, we integrated eQTL analysis with non-coding gene research to gain an insight into the genome-wide *cis*-action of variants on lncRNA expression throughout the 10 brain regions and highlighted its significance in human CNS disorders. We applied an enhanced probe set re-annotation strategy to quantify 11 587 lncRNAs in 1231 samples from 134 European-descent individuals without neurological disorders and conducted the *cis*-eQTL analysis in combination with the genotype data of 6 325 551 variants from the same individuals. Comparison with the GTEx dataset validated our findings and identified novel elncRNAs and eSNPs. The significantly lower expression levels of our novel elncRNAs compared to GTEx’s eGenes suggest that their identification may benefit from microarray technology: microarray probes can be optimized for sensitive detection of low-abundance transcripts, while RNA-seq typically requires greater sequencing depth to achieve comparable sensitivity. As shown in the above results, the elncRNAs exhibit higher expression levels and more complex expression patterns than the non-elncRNAs that are unlikely to be regulated by genomic variants across the brain. On the other hand, eSNPs have distinct characteristics compared to the non-eSNPs that do not affect lncRNA expression: (1) shorter distance to the TSS of the corresponding lncRNAs, (2) stronger enrichment among the variants located in non-coding and regulatory element genomic regions, and (3) higher MAF, across the whole brain. Moreover, the genetic regulation of lncRNA expression demonstrated greater tissue specificity compared to that of coding genes. The potency of genetic regulation on expression and the distribution and feature of elncRNAs exhibit differ markedly between the cerebrum and cerebellum while displaying the highest similarity among the cerebral cortex regions. This may be attributed to cerebellum’s highly homogeneous structure. With >70% of its neurons being granule cells, the cerebellum provides a more uniform environment, which likely leads to better-resolved datasets. This enhanced resolution offers a clearer view of SNP-regulated lncRNAs [50]. Moreover, gene expression data comparing the cerebellum with other brain regions, such as the prefrontal cortex, have revealed notable skewing. In studies of both humans and primates, the cerebellum consistently accounts for the majority of the gene expression changes, further supporting the cerebellum’s unique regulatory features [51]. Additionally, we found that genomic variants affecting lncRNA expression in the brain play a critical role in disease pathogenesis, especially neurological disorders. Furthermore, we found that the proximal localization of lncRNAs and mRNAs on the same chromosome further enhances their regulatory impact, providing additional support for a *cis*-regulatory mechanism of action for lncRNAs. This is likely underpinned by the enrichment of most lncRNAs in the chromatin fraction—specifically tethered to chromatin, presumably at their transcription sites via Pol II—and by their generally low expression levels, often only a few molecules per cell, which naturally favor *cis*-action due to limited diffusion or transport to distal cellular compartments [52–54]. A novel approach based on 2SLS algorithm was developed to explore the biological functions of the predicted insomnia-related lncRNAs. Also, we identified the immune-related pathways that could elucidate aspects of the mechanism underlying insomnia.

In conclusion, this study systematically analyzed the regulation of lncRNA expression based on genomic variants in the entire human brain. It offers valuable insights into the functional characterization of elncRNAs, their corresponding eSNPs, and their significance in human CNS disorders. In this process, we offered the following: (1) an improved probe set re-annotation method, enabling efficient utilization of the extensive microarray data resources already available; (2) a novel approach for the functional explanation of lncRNAs; (3) a database for storage and display of lncRNA expression regulation data in the human brain. These findings and methodologies serve as valuable resources for advancing the research on the regulation of non-coding gene expression in neuroscience.

## Methods

### Probe set re-annotation and lncRNA quantification

We obtained 1231 Affymetrix Exon 1.0 ST array CEL files (GEO: GSE60863) from 134 neuropathology-free European donors. Array quality control was performed using Expression Console (v1.4.1) with spike-in probes. LncRNAs were identified and quantified using an improved probe re-annotation strategy, followed by RMA normalization.

### *Cis*-eQTL analysis of lncRNAs

First, we converted genotype coordinates from hg19 to hg38 using dbSNP annotations [55]. Following quality control, 6 446 207 variants were retained for analysis. We then performed genome-wide *cis*-eQTL mapping of lncRNAs across 10 brain regions using Matrix eQTL, testing variants within ±1 Mb of transcription start sites. The analysis incorporated age, sex, three genotype principal components, and 15 PEER factors as covariates [56, 57]. Significant eSNPs were identified through permutation-based false discovery rate correction (FDR *q* < 0.05) [58]. Detailed quality control steps and analysis parameters are provided in Supplementary Methods.

### Differential expression and feature analysis of elncRNAs

First, a differential expression analysis was conducted between each brain region and the remaining regions using the R package limma [59]. The significance threshold was set at fold-change (FC) > 1.5 and FDR *q* < 0.05. The two-tailed Fisher’s exact test was used to compare the proportion of differentially expressed lncRNAs in the 440 elncRNAs versus the total 11 587 lncRNAs quantified. To compare the distributions of the 440 elncRNAs across the 10 brain regions, we used the Jaccard index (intersection of elncRNAs of two brain regions divided by their concatenation).

For the comparison of lncRNA features, a two-tailed Wilcoxon test was used to assess the differences in features between the elncRNAs from distinct brain regions. The lncRNA features were obtained from the LnCompare database [60]. Finally, a two-tailed Wilcoxon test was applied to compare the average expression and fluctuations in expression between elncRNAs and non-elncRNAs in each brain region. Expression *Z*-scores of the lncRNAs used for eQTL analysis were also calculated to evaluate their expression levels and fluctuations. LncRNAs with absolute median *Z*-scores greater than two in at least one individual were defined as outliers. The hypergeometric distribution test was used to assess the overlap between elncRNAs and the outliers in each brain region.

### eSNP profiling and functional characterization

To assess the specificity of genetic regulation on lncRNA expression in different brain regions, the genotyping data were annotated by ANNOVAR software against the refGene file (hg38) [61], and LD filtering was performed using PLINK with the 1000 Genomes Project phase 3 European data (threshold of *r*^2^ > 0.8) [62, 63]. Independent variants in *cis* regions with nominal *P* > .5 were defined as non-eQTL SNPs. Functional annotation of these variants was conducted based on 11 functional classes (including ncRNA intronic, ncRNA exonic, ncRNA splicing, upstream, downstream, 5′-UTR, 3′-UTR, exonic, intronic, splicing, and intergenic regions [64]). ChIP-seq peak data for human TF binding sites from ReMAP were used to explore the enrichment of eSNPs in regulatory elements. Fisher’s exact test was employed to evaluate the enrichment of eSNPs in TF binding sites. Additionally, the tissue specificity of genetic regulation on lncRNA expression across brain regions was assessed using an unbiased estimation algorithm to compare the correlation of beta values (${r}_b$) [40, 65].

### Variant frequency and eSNP analysis

To examine the relationship between allele frequency and regulatory impact, we analyzed MAF distributions of LD-pruned eSNPs versus non-eSNPs. All *cis*-SNPs were stratified into 5% MAF bins (5%–10%, 10%–15%, etc.), with variants below 5% MAF excluded during quality control. MAF calculations based on genotype dosage showed consistency with 1000 Genomes European population data. Then, we (1) calculated eSNP/non-eSNP proportions per MAF bin across 10 brain regions, (2) performed linear regression to assess frequency trends, and (3) used Fisher’s exact tests (FDR < 0.05) to compare distributions.

### Risk loci enrichment analysis of *cis*-eQTLs in disease

To assess the potential contribution of non-coding genetic regulation of lncRNAs to disease risks, we conducted a risk loci enrichment analysis in various human diseases using the association hits (*P* < 1 × 10^−5^) from the GWAS Catalog. The diseases were categorized according to the 11th edition of the International Classification of Diseases [66]. We selected a total of 94 neurological diseases and 417 non-neurological diseases from the GWAS Catalog [67]. Disease-related SNPs were defined as those in LD with the genome-wide significant index SNPs by PLINK using the 1000 Genomes Project phase 3 European data (*r*^2^ > 0.8) [62, 63]. Each disease was associated with an average of approximately 1281 SNPs (Supplementary Table S10). We then compared the proportions of LD-pruned eSNPs and non-eSNPs in each of the disease-related SNPs among the 10 brain regions using the two-tailed Fisher’s exact test with an FDR threshold of *q* < 0.05, adjusted by the Benjamini–Hochberg method.

### Identification and functional analysis of insomnia-related lncRNAs

First, we utilized eQTL data to train the TWAS prediction model of lncRNAs using PredictDB [68–70], and then combined it with a large-scale GWAS dataset (*n* = 1 331 010) to identify insomnia-related lncRNAs in the 10 brain regions. Second, since lncRNAs exert their functions by regulating mRNA expression, we designed an approach based on 2SLS estimation to discern this causal influence by leveraging eQTL data. Protein-coding genes were quantified in the same samples used for lncRNA quantification [71]. Finally, based on the affected mRNA, we employed enrichment analysis to deduce the functions of insomnia-related lncRNAs using the R package clusterProfiler [72]. The GO dataset was used as the reference, and the following criteria were applied: TWAS *q* < 0.1 and number of affected mRNAs > 20 to select the lncRNAs of interest.

**Key Points**

- This comprehensive multi-omics study established a refined regulatory atlas of lncRNA expression, uncovering novel eSNPs and elncRNAs across 10 distinct brain regions, and highlighting pronounced tissue-specific regulatory patterns.
- Common variants with higher minor allele frequency are enriched as eSNPs, suggesting their key role in regulating lncRNA expression in the human brain.
- The eSNPs show significant enrichment in neurological disorders, particularly insomnia, with related lncRNAs linked to immune response functions.
- Improved methods for lncRNA identification and quantification, functional interpretation, and a database for brain lncRNA expression regulation were offered (http://lncbrain.org.cn/ and https://relncann.cqmu.edu.cn/appcel/).

## Supplementary Material

Han-Supplementary_Figures_bbaf291

Han-Supplementary_Tables_S1_to_S16_bbaf291

Han-Supplementary_Methods_bbaf291

## Data Availability

The datasets of lncRNA expression, *cis*- and *trans*-eQTL, trained lncRNA prediction models and covariance matrices for TWAS in the 10 brain regions, and the LD results of variants in *cis*-eQTL region can be accessed and downloaded from the lncBRAIN database (http://lncbrain.org.cn/). Access to the other original data needs to be requested from the United Kingdom Brain Expression Consortium (UKBEC). The tools for quantifying lncRNA s by probe set re-annotation method are stored in the web server RElncANN (https://relncann.cqmu.edu.cn/appcel/). The codes used in this study are available at https://github.com/hyj260/LncBRAIN.
