# Supplementary material for: Genetic regulation of lncRNA expression in whole human brain and their contribution to CNS disorders
Source: Brief Bioinform. 2025 Jun 21;26(3):bbaf291. doi: 10.1093/bib/bbaf291 (PMC12205935; doi:10.1093/bib/bbaf291)
Supplement: Han-Supplementary_Figures_bbaf291 [file han-supplementary_figures_bbaf291.docx]

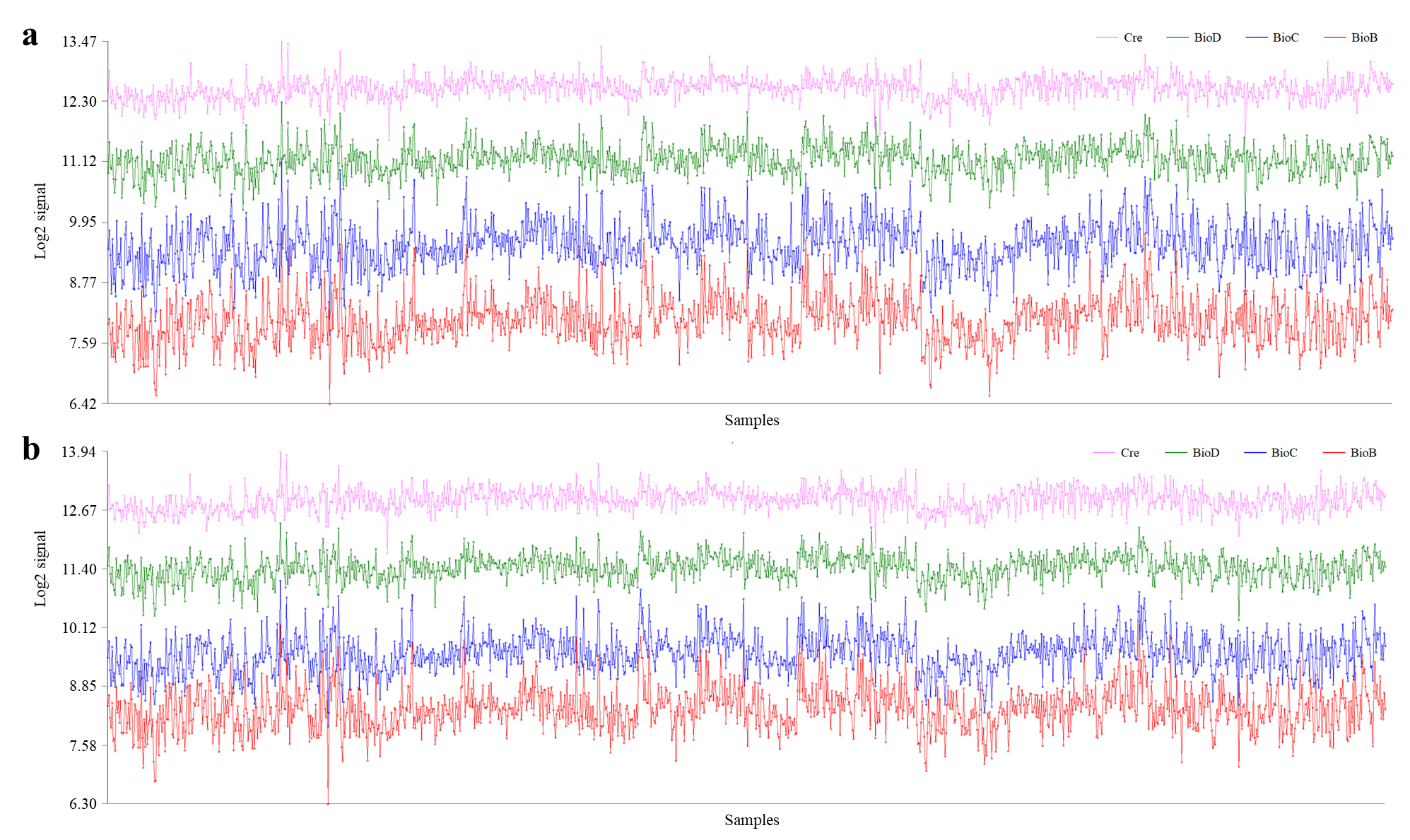


**Figure S1** Quality control of the Affymetrix Exon 1.0 ST arrays by the hybridization concentration assessment. The pre-labeled exogenous spike-in probes are BioB, BioC, BioD and Cre from 5’ site (**a**) and 3’ site (**b**), respectively.


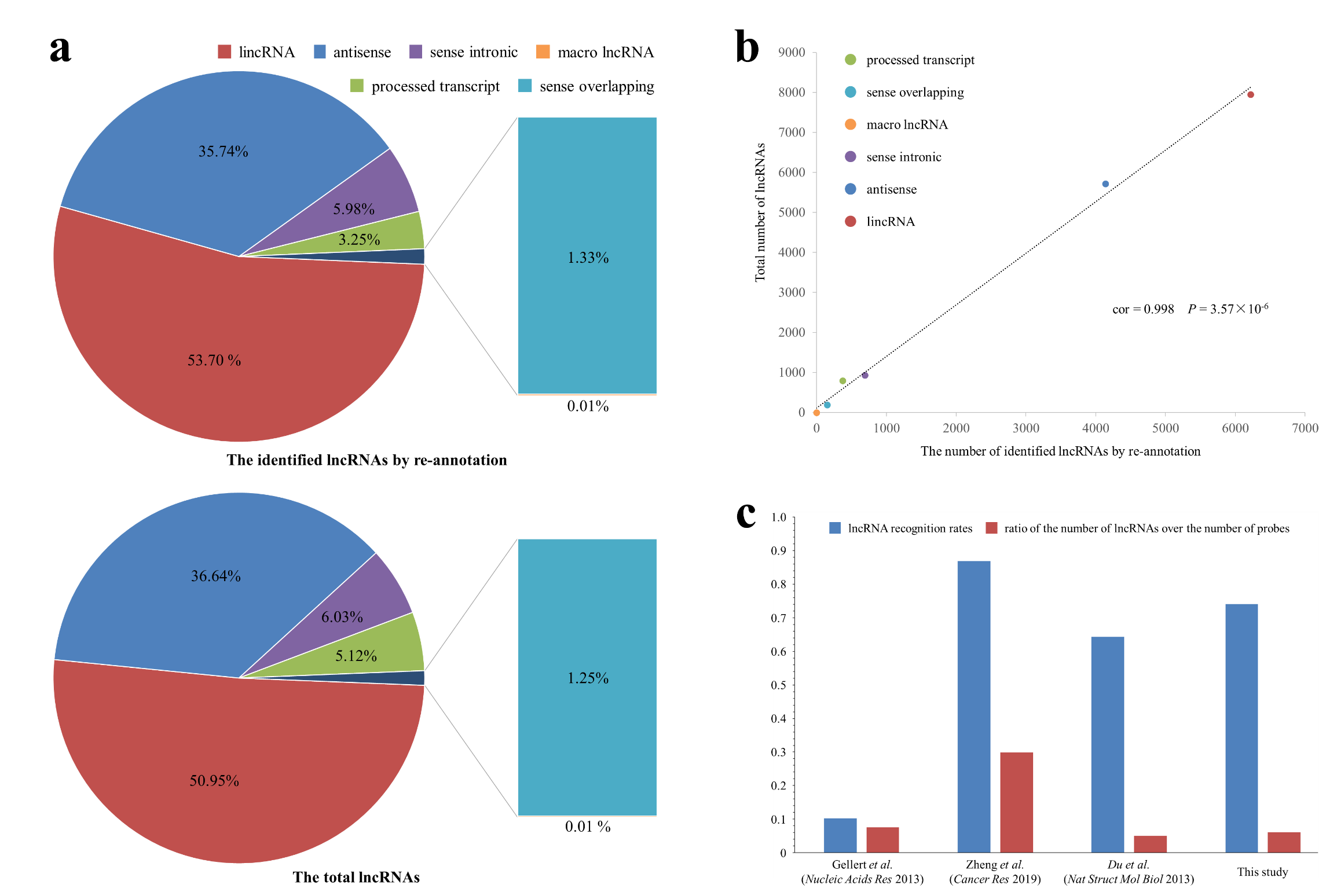


**Figure S2** Affymetrix Exon 1.0 ST arrays probe set re-annotation for identifying lncRNAs. (**a**) Percentage of the six subtypes in the identified lncRNAs by re-annotation and the total lncRNAs, respectively. (**b**) Consistency of the proportions of the six subtypes in two groups. (**c**) Overall advantage of the improved probe set re-annotation approach used in this study compared with the previous methods.


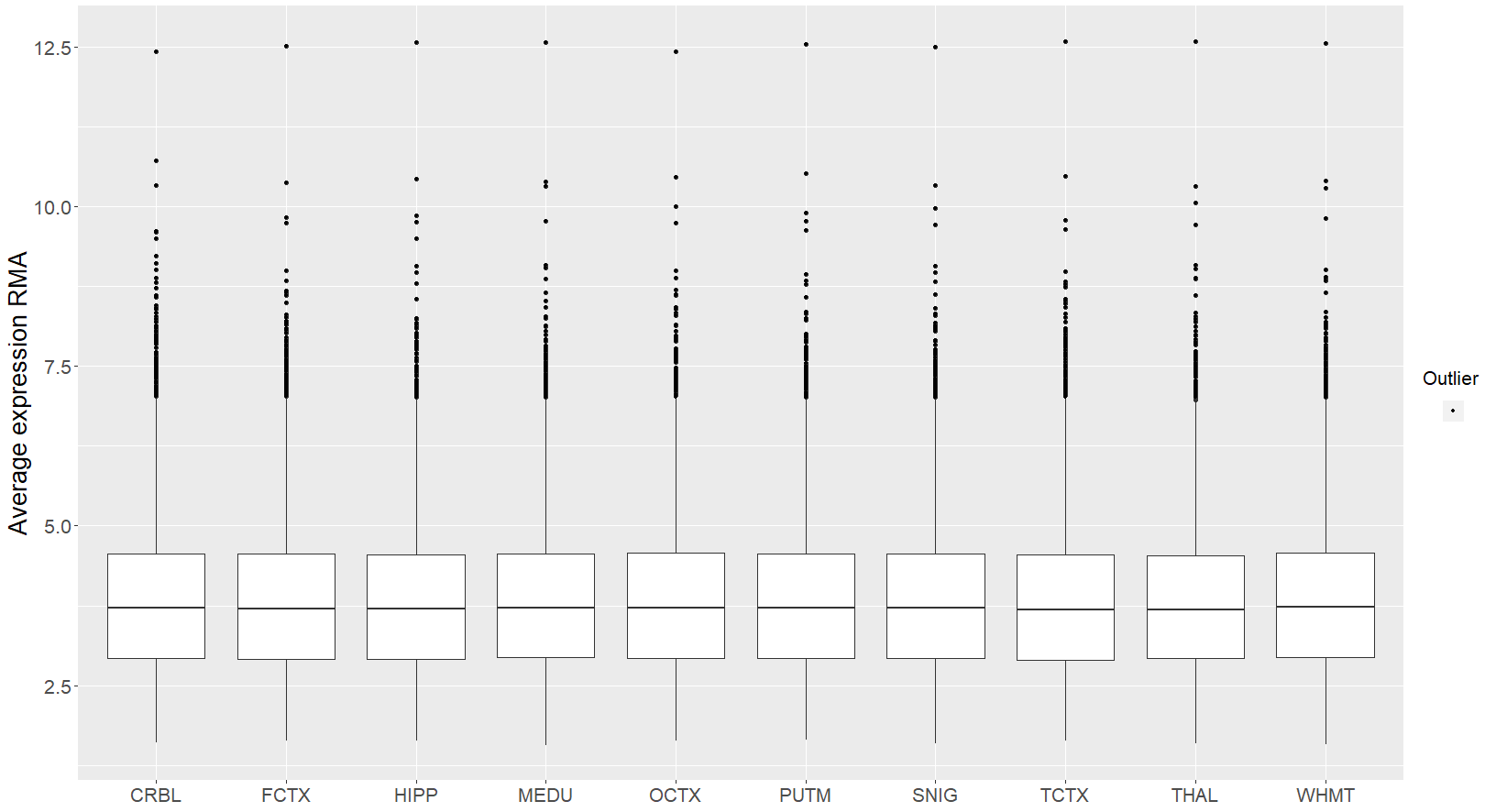


**Figure S3** The overall expression level of the 11,587 lncRNAs identified by exon arrays probe set re-annotation across the 10 brain regions. Each element represents the average expression of a lncRNA in a brain region.


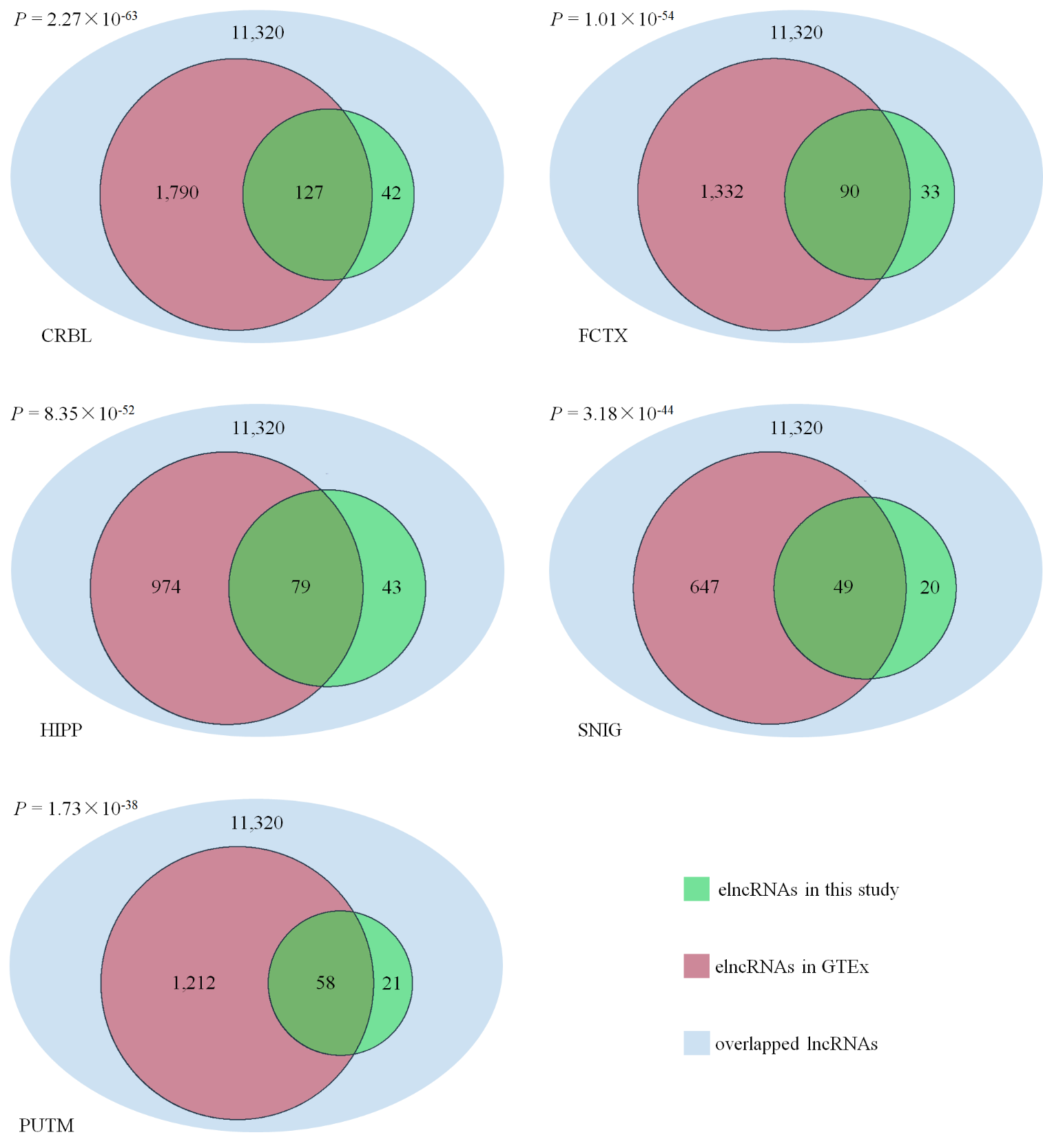


**Figure S4** The replication of our identified elncRNAs in brain region CRBL, FCTX, HIPP, SNIG, and PUTM in GTEx (v8) datasets. The statistical significance was determined by hypergeometric distribution test with the 11,320 overlapped lncRNAs as the background.


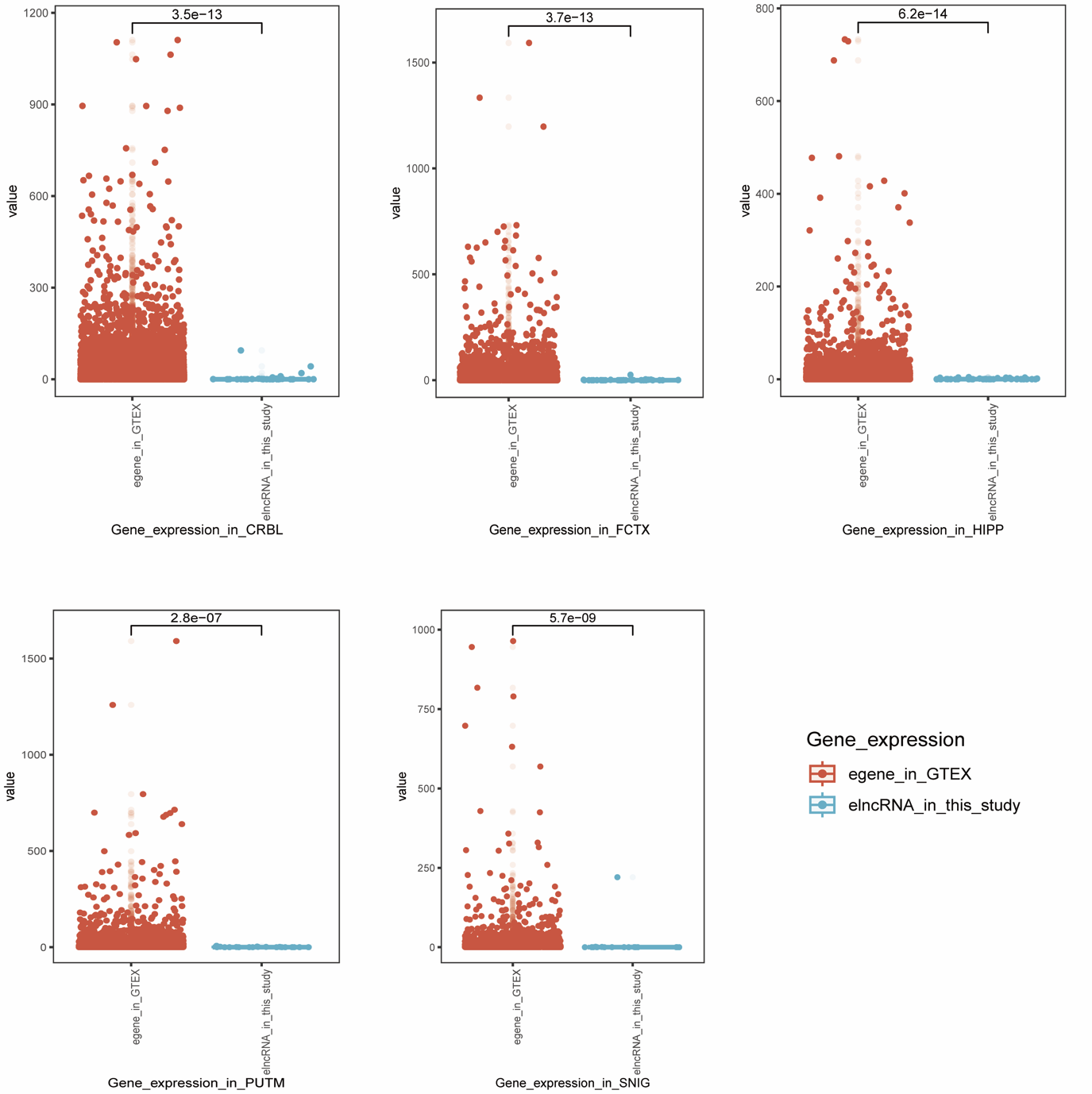


**Figure S5** Expression differences between study-specific elncRNAs and GTEx-exclusive eGenes in five brain regions (CRBL, FCTX, HIPP, SNIG, PUTM), analyzed by Wilcoxon test.


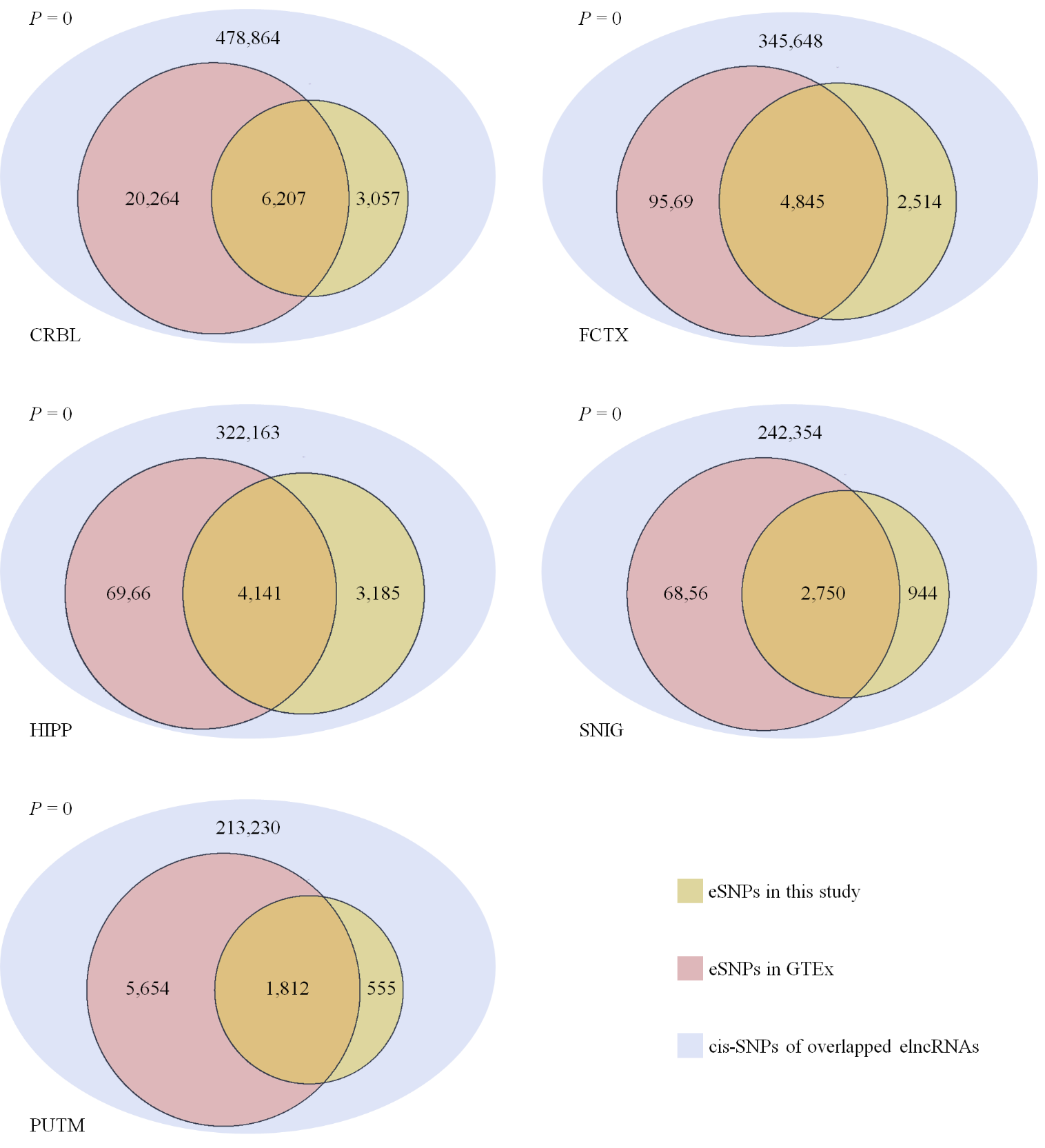


**Figure S6** The replication of our identified eSNPs in brain region CRBL, FCTX, HIPP, SNIG, and PUTM in GTEx (v8) datasets. The statistical significance was determined by hypergeometric distribution test with cis-SNPs of overlapped elncRNAs in each brain region as the background, respectively.


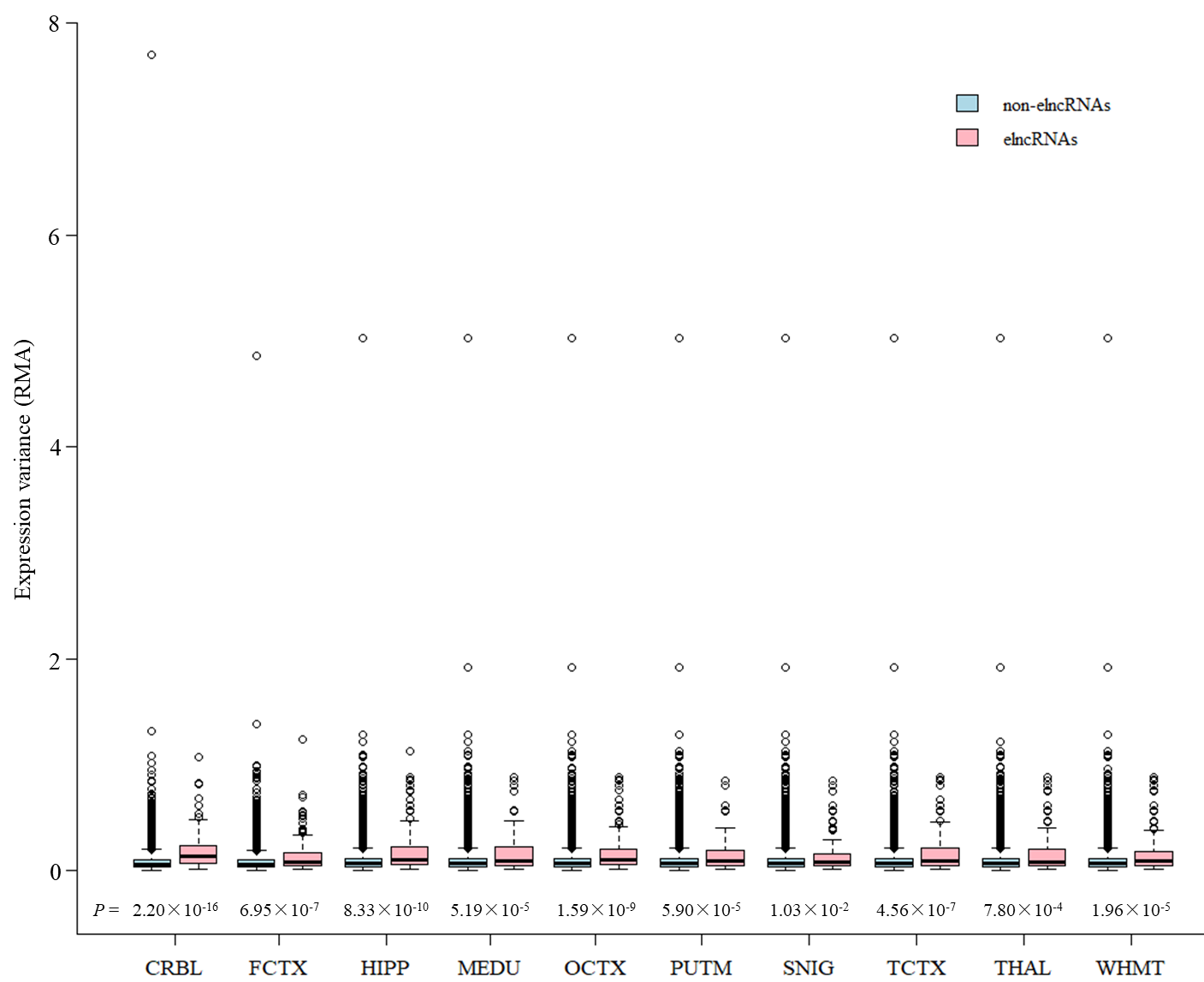


**Figure S7** The box plots show the expression variance of each elncRNA and non-elncRNA across individuals in the ten brain regions. The *P* values were calculated by two-tailed Wilcoxon test.


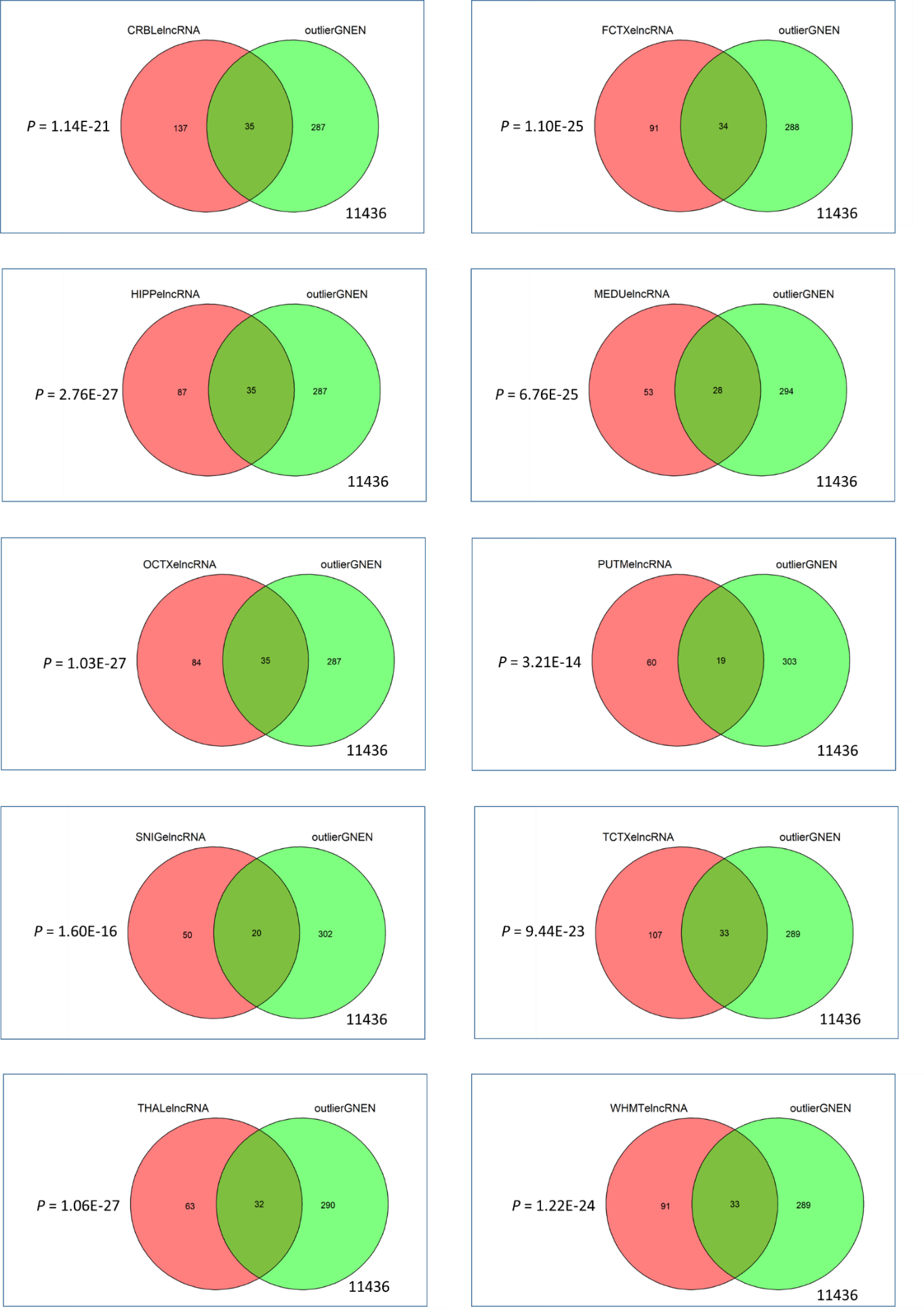


**Figure S8** The overlap between elncRNAs and outliers in each brain region. The statistical significance was determined by hypergeometric distribution test with the 11,436 lncRNAs used for eQTL analysis and outlier discovery as the background.


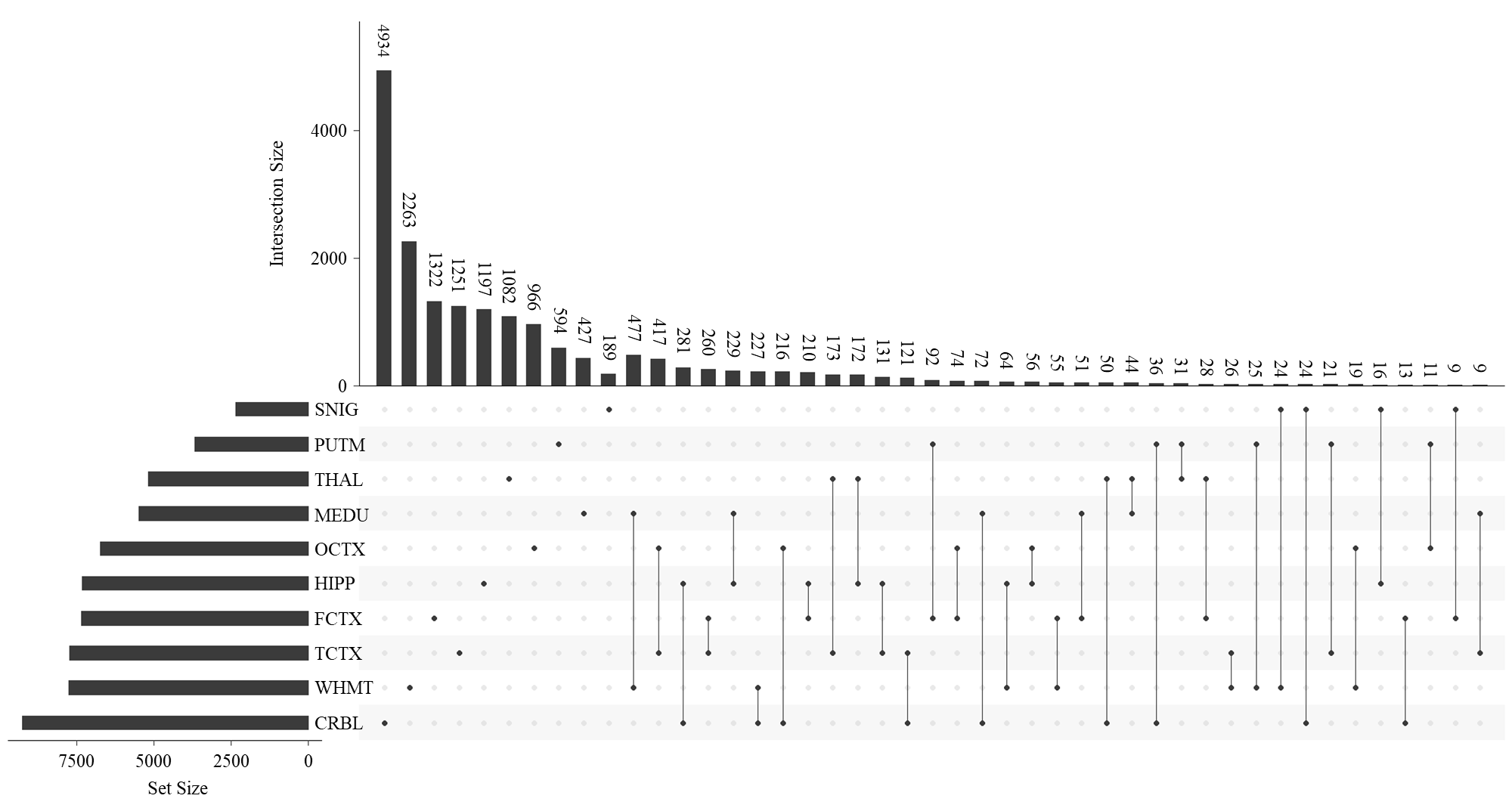


**Figure S9** The UpSet plot showing the number of eSNPs obtained from different brain regions and their tissue specificity and intersection.


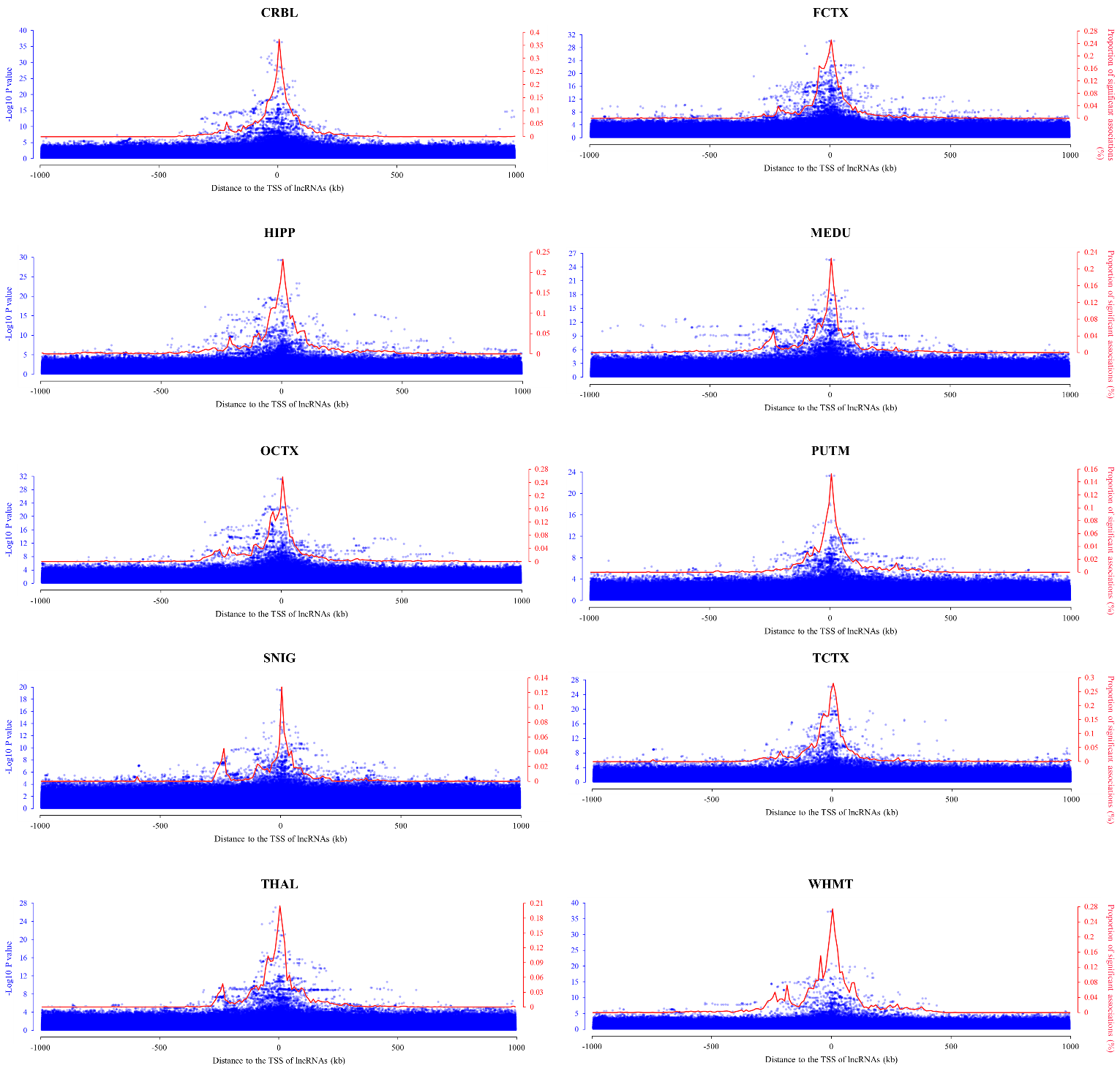


**Figure S10** Significance of eQTLs and percentage of eSNPs in relation to the distance from variants to TSS of corresponding lncRNAs in the ten brain regions. The blue dots represent the variants in cis-regions with the negative log-transformed eQTL P values (left y-axis). The red line represents the percentage of eSNPs per 10 kb window (right y-axis).


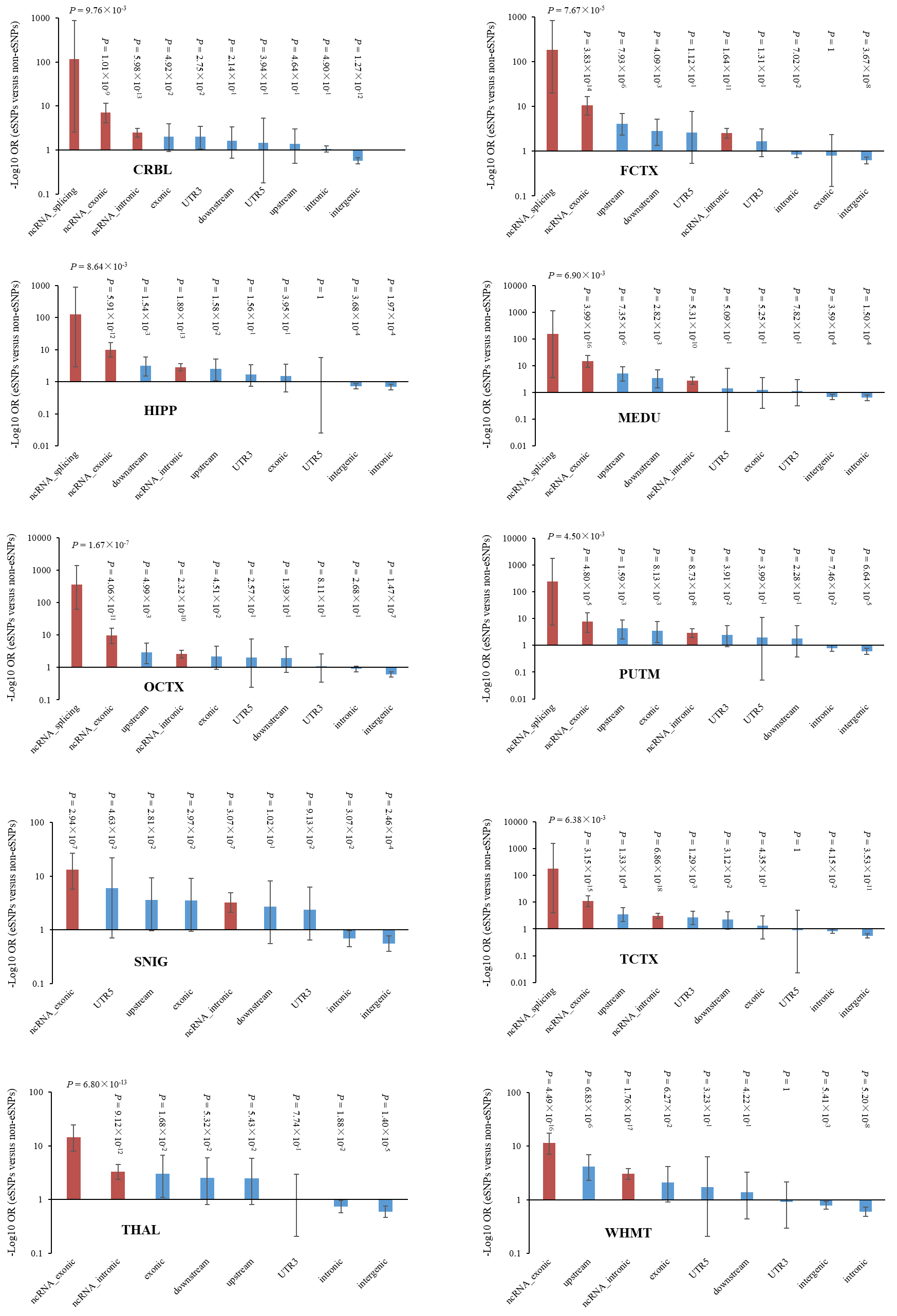


**Figure S11** Enrichment analysis of the eSNPs among different functional categories of variants in the ten brain regions measured by the two-tailed Fisher’s exact test. The variants in non-coding genomic regions were marked red and others were marked blue. The black bars in histogram indicate 95% CI.


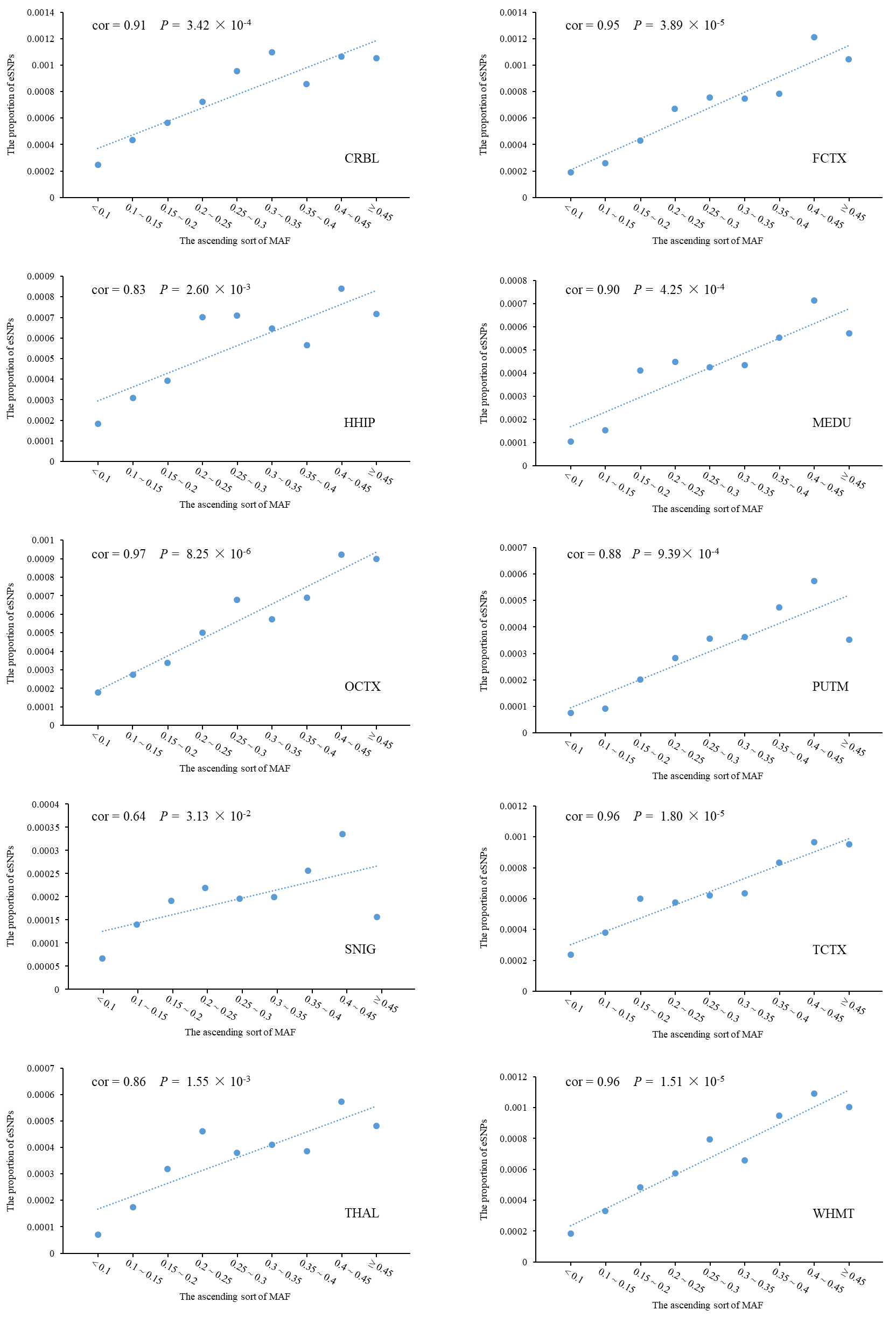


**Figure S12** The change in proportion of eSNPs among cis-SNPs with increasing MAF in the ten brain regions. The blue points represent the proportions in corresponding MAF bin, and the dotted lines are their linear regression results.


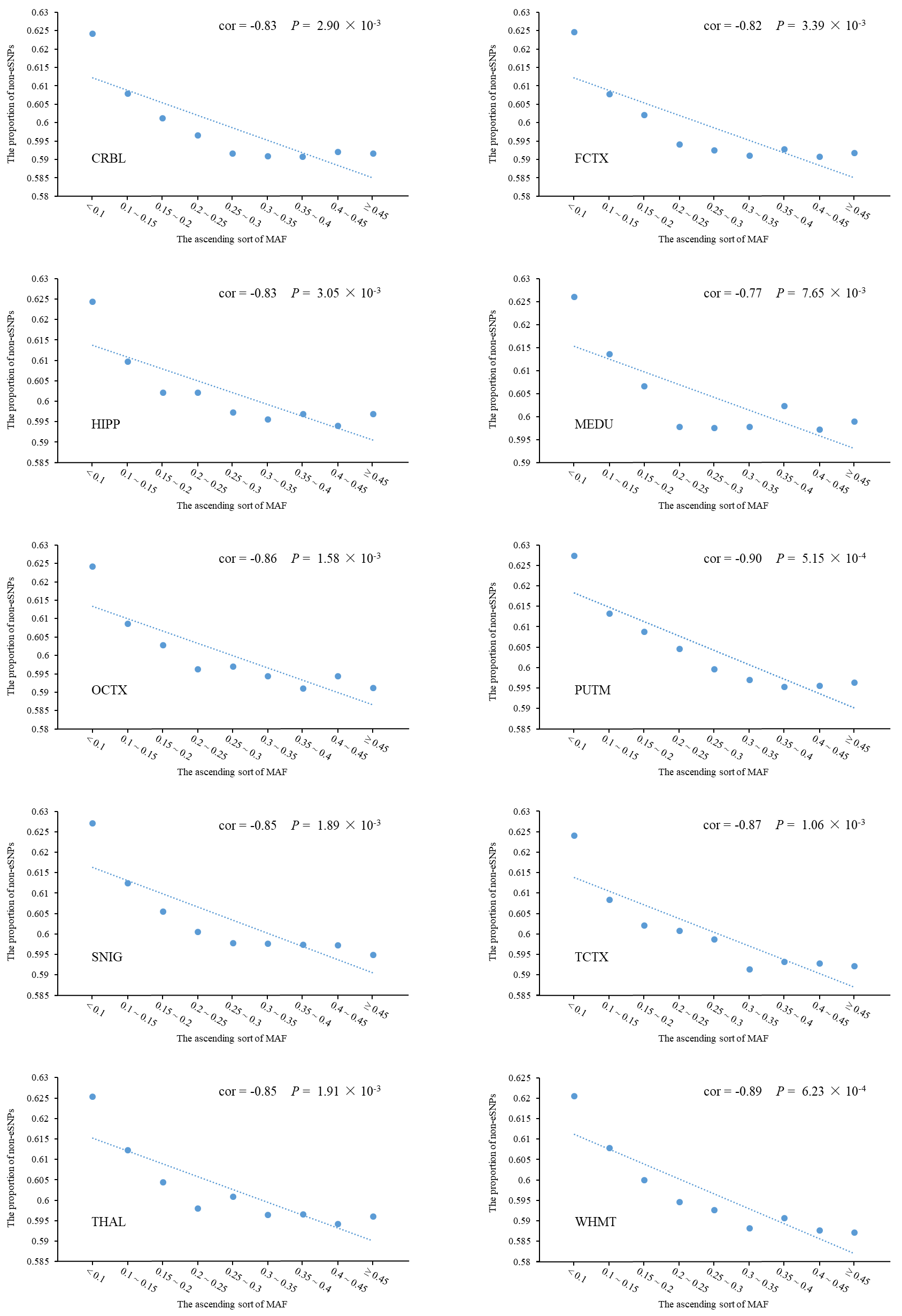


**Figure S13** The change in proportion of non-eSNPs among cis-SNPs with increasing MAF in the ten brain regions. The blue points represent the proportions in corresponding MAF bin, and the dotted lines are their linear regression results.


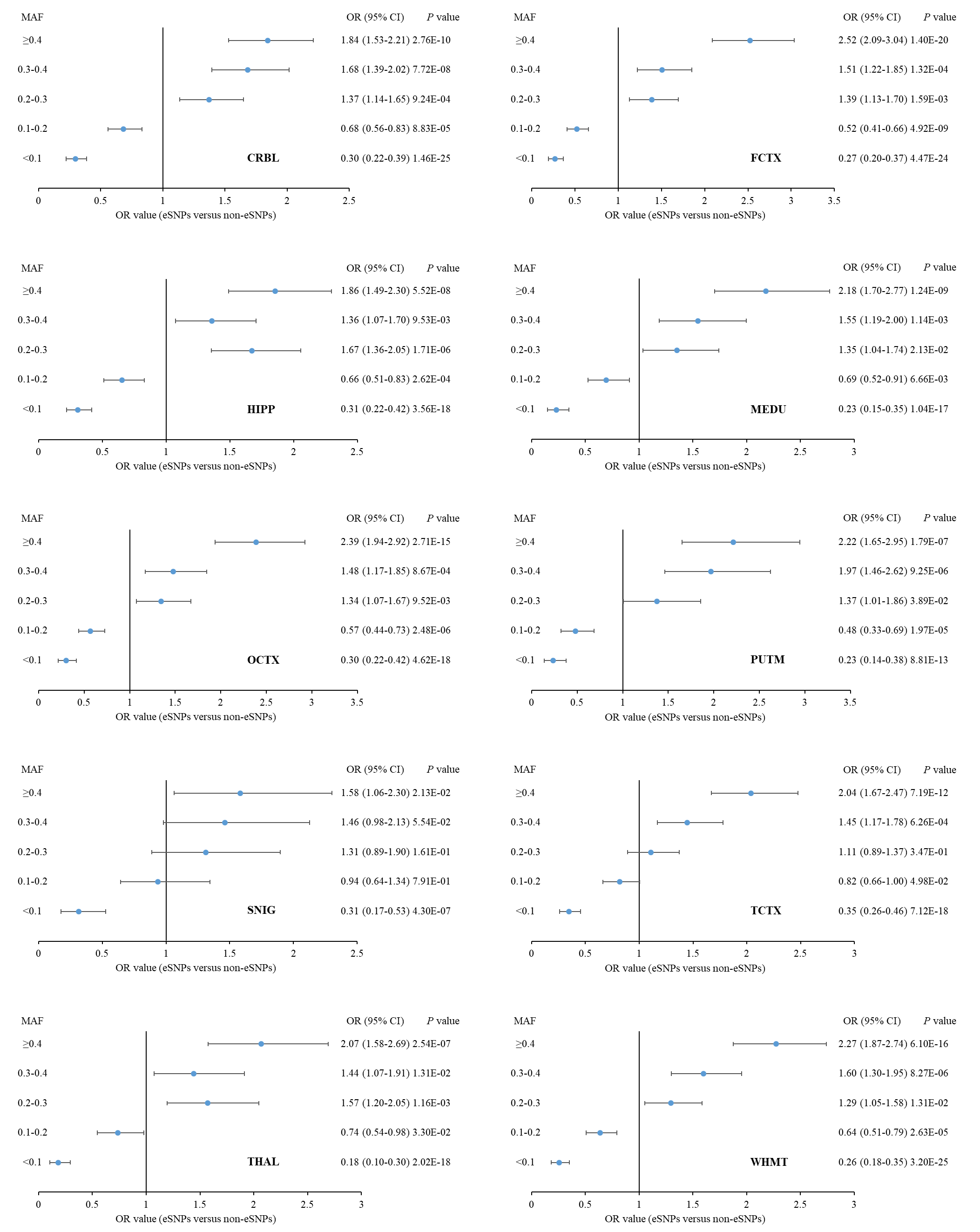


**Figure S14** The forest plot showing the enrichment results of eSNPs when compared with the non-eSNPs in each MAF bin of each brain region. The OR and 95% CI were calculated by two-tailed Fisher’s exact test.
