# Supplementary material for: Genetic regulation of lncRNA expression in whole human brain and their contribution to CNS disorders
Source: Brief Bioinform. 2025 Jun 21;26(3):bbaf291. doi: 10.1093/bib/bbaf291 (PMC12205935; doi:10.1093/bib/bbaf291)
Supplement: Han-Supplementary_Methods_bbaf291 [file han-supplementary_methods_bbaf291.docx]

**Supplementary Methods**

**Genotype and microarray data from ten brain regions of 134 individuals**

A total of 1,231 samples (ten brain tissues) of 134 European-descent individuals free of neurological disorders from UKBEC were used in this study [1]. Briefly, the postmortem brains were selected from two sources: the Medical Research Council Sudden Death Brain of Edinburgh and the Sun Health Research Institute Brain Bank of USA. The average age of these individuals at the time of death was about 58 years (ranging from 16 to 102), the mean sample size per brain region was about 123 (ranging from 101 to 131), and the sex ratio (female to male) of these individuals is about 0.35 (**Supplementary Table S1**). Then, for transcriptome profiling, total RNA was extracted from each brain tissue using a single-step RNA isolation method and the miRNeasy 96 kit. After the quality control checks on the Agilent 2100 Bioanalyzer and RNA 6000 Nano Kit, 1,231 qualified samples were processed on 96-well plates at microarray facility and hybridized to Affymetrix Exon 1.0 ST arrays. Subsequently, the arrays were scanned and inspected visually using Affymetrix GeneChip Scanner 3000 7G. The Affymetrix Exon 1.0 ST array CEL files were stored in the GEO database with the accession code GSE60863. Finally, for genotype profiling, genomic DNA was isolated from each brain tissue using DNeasy Blood & Tissue Kit (Qiagen) and subjected to genotyping using Illumina Infinium Omni1-Quad BeadChip and Immunochip. Then, the array scan was carried out on an Illumina iScan system, followed by SNP calling and imputation using Illumina GenomeStudio and MaCH/minimac, respectively. The variants with MAF < 5% and low-quality genotype data were removed. The filtering criteria included the samples with < 95% call rate, suspected non-European ancestry or cryptic sex status and relatedness, variants with false allele or heterozygote, missing genomic location info, genotyping call rate < 95% or Hardy-Weinberg equilibrium (HWE) P value < 0.0001. The more detailed description of sample and upstream analysis is provided in Ramasamy *et al.* [1]. Furthermore, we converted the genomic coordinates of these variants (hg19) into the GRCh38 human genome (hg38) according to the annotation in dbSNP (version 151, human 9606) [2]. Finally, the mismatched variants were removed to obtain a total of 6,446,207 genotyped variants (hg38) were obtained for the following analyses.

**Probe set re-annotation and lncRNA quantification**

The previous probe set re-annotation methods were improved to identify and quantify lncRNAs from the Affymetrix Exon 1.0 ST arrays [3-5]. Specifically, the Ensembl gene annotation file with the corresponding reference sequence (Homo sapiens GRCh38/hg38, release 82) was used for the probe set re-annotation and lncRNA identification. It includes 15,618 lncRNAs, 22,017 protein-coding genes, 28,406 pseudogenes and other non-coding genes [6]. After removing the quality control probes, the remaining 5,383,654 probes were mapped to the reference sequence with no mismatch using SeqMap software [7]. Based on the Ensembl sequence annotations, we removed the two types of unsuitable probes: (1) those mapping to the protein-coding genes, pseudogenes, or other non-coding genes; (2) those multi-mapping to more than one gene, whereas those mapping to multiple transcripts belonging to same gene were retained. According to the principle of matching at least four probes per lncRNA, we identified 11,587 lncRNAs and 189,164 corresponding eligible probes. Next, array quality control was performed using the Expression Console software (v 1.4.1) based on quality control probes. The array hybridization and overall signal quality metrics were determined using the pre-labeled exogenous probes with an increasing hybridization concentration, i.e., the BioB, BioC, BioD and Cre signals are arranged in order from lowest to highest (**Supplementary Figure S1**). Finally, based on the lncRNA probe set re-annotation file, the basicRMA function of R package oligo with default parameters was utilized to normalize raw background intensity of array probes via the RMA algorithm and to quantify the expression level of these identified lncRNAs in the 1,231 samples [8].

**Genome-wide cis-eQTL analysis of lncRNAs**

First, we removed 121 lncRNAs, not annotated with genomic coordinates by Ensembl (GRCh38, release 82), from the 11,587 quantified lncRNAs (**Supplementary Table S2**). Then, the genomic locations of all the 6,325,551 genotyped variants were mapped to a 1 Mb region upstream and downstream of the remaining 11,466 lncRNA TSSs (cis regions) based on the dbSNP annotation (version 151, human 9606) [2]. After removing 30 lncRNAs that did not contain a variant in the cis regions, we selected 6,325,551 variants within 1 Mb around the TSS of 11,436 lncRNAs. Subsequently, a cis-eQTL analysis was conducted in the ten brain regions using the R package Matrix eQTL, respectively, based on the RMA values of the 11,436 lncRNAs in combination with the genotype data of the 6,325,551 variants [9]. A linear regression model was used in the eQTL analysis with parameters age, gender, first three PCs, and first 15 PEERs serving as covariates. Specifically, we used the “transpose trick” in principal component analysis (PCA) to generate the first three PCs of genotype data (high dimensionality). This approach could circumvent a large memory footprint by directly computing the covariance matrix without forming the transpose of the centered data matrix. Factor analysis and Bayesian regression modules were applied to generate the first 15 PEERs from the lncRNA expression data using the R package PEER with parameters age and gender serving as covariates [10]. Subsequently, a permutation procedure was employed to determine the elncRNAs without multiple hypothesis effects of variants in LD, which were used as a basis for the subsequent eSNP discovery, as described previously [11, 12]. Briefly, the minimum nominal *P* value corresponds to the most significant result of cis-eQTL analysis per lncRNA. The permuted *P* values were calculated by randomizing the sample labels of lncRNA expression data and their related covariates (i.e., age, gender, and PEERs), while holding fixed the genotype data and corresponding covariate PCs. The permutations were conducted from a minimum of 1,000 to a maximum of 10,000 and exited when at least 15 minimum permuted *P* values were less than the minimum nominal *P* values. The empirical *P* value of per lncRNA was defined as the ratio of occurrences of extreme events (minimum permuted *P* < minimum nominal *P*) to the total number of permutations (N_p_). The multiple testing corrections of the empirical *P* value were performed using Storey approach, and the threshold of significant elncRNAs was set at FDR *q* < 0.05. Finally, a permutation threshold of each lncRNA was used to identify the eSNPs. The empirical *P* value of lncRNA whose FDR *q* value was closest to 0.05 (due to the nonlinear transformation of Storey approach) was selected to generate a percentile (*P* × N_p_ of a specific lncRNA), and the permuted *P* value at this percentile (ascending order and ceiling integer) was defined as the permutation threshold for each lncRNA in each brain region. An eSNP was determined if its nominal *P* value was less than the threshold value.

**Differential expression analysis**

The differential expression of elncRNAs and total quantified lncRNAs between each brain region and the remaining regions was analyzed, respectively. Firstly, the lmFit function of the R package limma was used to fit a linear model of the expression matrix. Then, the estimated t-statistic and FCs of lncRNAs between each brain region and the remaining regions were calculated by the contrasts.fit function based on the fitted model. Next, the eBayes function was applied to moderate the FCs and to generate the Bayes factors. Finally, the Benjamini-Hochberg adjustment was used for multiple tests based on the ranking of Bayes factors. All functions were executed using the default parameter settings. According to the significance threshold of FC > 1.5 and FDR *q* < 0.05, we identified 116 elncRNAs (**Supplementary Table S6**) and 908 lncRNAs (**Supplementary Table S7**) with differential expression.

**Comparison of lncRNA expression and features across brain regions**

Herein, we used the two-tailed Wilcoxon test to assess the differences in lncRNA expression, characteristic features, and the number of cis-SNPs. Wilcoxon test is a non-parametric statistical test appropriate for the normal or non-normal distribution data. Specifically, a comprehensive feature set of lncRNAs based on the human GENCODE annotation (v24) is cataloged in the LnCompare database, which includes lncRNA length, classification, localization, sequence conservation, nucleotide composition, and transcriptional level in specific cell lines and tissues [13]. Firstly, these features were normalized from 0 to 1 using the R package scales (<https://CRAN.R-project.org/package=scales>), respectively. Then, the distribution of each normalized feature of elncRNA sets was compared between the pairs of brain regions using the R function wilcox.test with the default parameters. The lncRNAs with empirical *P* > 0.05 were defined as the non-elncRNAs, and the distribution of their average expression levels and variances were compared across individuals per brain region with the elncRNAs by the two-tailed Wilcoxon test, respectively. Finally, we selected the SNPs located in the cis region of the lncRNAs common to this study and GTEx (used for the eQTL analysis) from the two studies, respectively, and compared their number using the two-tailed Wilcoxon test. The significance threshold of the Wilcoxon test was set at *P* < 0.05.

**Statistical analysis of lncRNA characteristics and variant associations**

The two-tailed Fisher’s exact test was conducted with a 2 × 2 table in this study for the elncRNA characterization analysis (rows of 2 × 2 table: differentially expressed and non-differentially expressed lncRNAs, columns: elncRNAs and total lncRNAs), type enrichment analysis of eSNPs (row: SNPs within and not within a type, columns: eSNPs and non-eSNPs), TF binding site analysis (row: SNPs within and not within regions of TF peaks, columns: eSNPs and non-eSNPs), comparison of variant frequency (row: SNPs within and not within a MAF bin, columns: eSNPs and non-eSNPs), disease association analysis (rows: disease- and non-disease-related SNPs, columns: eSNPs and non-eSNPs), comparison of SNP-enriched diseases (rows: proportion of SNP-enriched neurological and non-neurological diseases, columns: total neurological and non-neurological diseases), and disease-brain region pairs (rows: proportion of SNP-enriched neurological and non-neurological disease-brain region pairs, columns: total neurological and non-neurological disease-brain region pairs), respectively. These analyses were carried out using R function fisher.test.

**Analysis of overlap between eQTL, outliers, and TWAS results**

The hypergeometric distribution test was applied to demonstrate the overlap between the different sets using the R function phyper(k, m, N-m, n, lower.tail = FALSE). In the overlap of elncRNAs identified by GTEx and this study, N indicates the 11,320 lncRNAs shared by the eQTL analysis of GTEx and this study, m refers to the number of elncRNAs identified by GTEx, n indicates the number of elncRNAs identified by this study, and k refers to the number of elncRNAs shared in GTEx and this study from the corresponding brain regions. In the overlap of eSNPs identified by GTEx and this study, N refers to the number of shared SNPs used for cis-eQTL analysis of the overlapped elncRNAs in GTEx and this study, m indicates the number of the eSNPs identified by GTEx, n stands for the number of eSNPs identified in this study, and k refers to the number of the eSNPs shared in GTEx and this study from the corresponding brain regions. In the overlap of elncRNAs and outliers, N refers to the total of 11,436 lncRNAs used for eQTL analysis and outlier discovery, m indicates the number of elncRNAs, n stands for the number of outliers, and k refers to the number of elncRNAs in outliers from the corresponding brain regions. In the overlap of elncRNAs and TWAS results, N refers to the total 11,436 lncRNAs used for eQTL analysis and TWAS analysis, m indicates the number of elncRNAs, n refers to the number of TWAS findings, and k stands for the number of elncRNAs in TWAS findings from the corresponding brain regions. The significance threshold of the hypergeometric distribution test was set at *P* < 0.05.

**LncRNA expression outlier discovery**

The lncRNA expression outliers were discovered using the protocol described previously [14]. Briefly, the standardized expression values of the 11,436 lncRNAs used for eQTL analysis were log2-transformed (i.e., log_2_(RMA+2)) before calculating their mean expression level in each brain region. Then, the mean expression was used as the constant term to regress out the gender, first three PCs, and top 15 PEERs from the corresponding lncRNA expression levels via a linear model using R function lm in each brain region. Furthermore, based on the assumption of normality of the residual vector, we standardized the expression residuals of each lncRNA and yielded their Z-scores in each sample using R function rstandard. Finally, the median of absolute Z-scores for each lncRNA across brain regions for each individual (the individuals with fewer than 5 brain regions were removed) was calculated, and the lncRNAs with absolute median Z-score > 2 at least in one individual were defined as the outlier.

**Generation of independent** **eSNPs and non-eSNPs as well as disease-related SNPs**

We identified both eSNPs and non-eSNPs in the cis regions of lncRNAs. The eSNPs were defined according to their nominal *P* value less than the permutation threshold of the corresponding lncRNAs (as described above). On the contrary, the non-eSNPs were defined based on their nominal *P* > 0.05 (much greater than the permutation threshold) for all lncRNAs, which are unlikely to be associated with their expression in the corresponding brain regions. Non-eSNPs were defined using a more lenient P-value threshold (P > 0.05) than eSNPs to exclude SNPs with any nominal association evidence, ensuring that non-eSNPs truly represent variants less likely to be associated with gene expression. This improves the reliability of downstream analyses by minimizing potential overlap or ambiguity between the two groups. Then, we performed an LD-based filtering procedure to remove the variants genetically dependent on each other from the eSNPs and non-eSNPs, respectively. Specifically, the *P* value-informed clumping was used to conduct the LD pruning by PLINK based on the 1000 Genomes Project phase 3 European data with the following parameters: window size of 250 kb and r^2^ threshold of 0.8. The number of independent eSNPs and non-eSNPs in the ten brain regions were 647 and 592,436 (CRBL), 548 and 592,683 (FCTX), 471 and 595,568 (HIPP), 345 and 597,597 (MEDU), 461 and 593,728 (OCTX), 245 and 597,813 (PUTM), 167 and 597,099 (SNIG), 544 and 594,139 (TCTX), 295 and 596,337 (THAL), and 550 and 590,628 (WHMT), respectively. Moreover, their numbers in the whole brain were 2629 (concatenation of eSNPs of the ten brain regions) and 1,927,671 (concatenation of non-eSNPs of the ten brain regions after removing eSNPs), respectively.

In order to generate multiple sets of disease-related SNPs, we first obtained the genome-wide significant index SNPs from the GWAS Catalog database according to the threshold of *P* < 1.0×10^-5^. GWAS Catalog provides summary results and comprehensive information about 276,696 index SNPs of 5,481 human diseases and traits [15]. ICD-11 is an international standard classification system of disorders (<https://icd.who.int/en>) comprising 26 chapters, each corresponding to a specific category of diseases [16]. Chapter 6 (mental behavioural or neurodevelopmental disorders), chapter 7 (sleep-wake disorders), and chapter 8 (diseases of the nervous system) were classified as the neurological diseases, and the remaining were termed non-neurological diseases. Of these, 511 (94 neurological and 417 non-neurological diseases) were selected and included in the GWAS Catalog. Finally, we used PLINK to extend the index SNPs on the 1000 Genomes Project phase 3 European data (window size = 1Mb, r^2^ > 0.8), and collectively defined the index SNPs and those in strong LD as the disease-related SNPs. A total of 159,358 neurological and 530,549 non-neurological disease-related SNPs were identified in different diseases (**Supplementary Table S10**).

**Variant functional annotation**

All the 6,446,207 genotyped variants were functionally annotated using ANNOVAR software, a Perl command-line tool designed for efficiently and rapidly annotating the genomic variant data derived from high-throughput analysis (such as VCF file) [17]. The refGene database (hg38) was used as the annotation reference file consisting of functional information of variants in a genome-wide scale. It categorizes the variants as the following classes: ncRNA intronic, ncRNA exonic, ncRNA splicing, upstream, downstream, 5’-UTR, 3’-UTR, exonic, intronic, intergenic, and splicing site [18]. After removing 1,553 variants that are not annotated accurately (for example, annotated with multiple functional classes), we obtained a total of 6,444,654 variants annotated with unique functional classes. For the LD-pruned variants, the independent eSNPs and non-eSNPs were annotated into ten (lack of splicing variants) and 11 classes, respectively. Specifically, the numbers of the annotated independent eSNPs and non-eSNPs in the ten brain regions and whole brain were 646 and 592,299 (CRBL), 547 and 592,517 (FCTX), 470 and 595,412 (HIPP), 345 and 597,429 (MEDU), 460 and 593,566 (OCTX), 245 and 597,663 (PUTM), 167 and 596,922 (SNIG), 543 and 593,956 (TCTX), 294 and 596,187 (THAL), 549 and 590,478 (WHMT), and 2,628 and 1,927,132 (whole brain), respectively. The detailed statistics are described in **Supplementary Table S16**.

**Selection of TF binding sites**

We downloaded the “brain-prefrontal-cortex 2022” (hg38) dataset was downloaded from a multiple-tissue TF ChIP-seq database ReMAP [19]. This dataset contains 133,084 peaks of TF SIN3A and OLIG2 binding sites in human FCTX, which is the only tissue that matched our study. Then, we selected the LD-pruned eSNPs and non-eSNPs of FCTX, and mapped them to the significant peak regions of the two TF binding sites by genomic coordinates, respectively.

**Correlation of** **lncRNA cis-****eQTL effects among brain regions.**

Herein, we compared the similarity of genetic regulation on lncRNA expression between two of the ten brain regions by calculating the $r_{b}$. It is an unbiased correlation estimate of eQTL effects, which accounts for both effect strength (beta values) and significance (errors or *P* values) in this process [20]. According to the previous study, we first selected the top-associated cis-eQTLs of the 425 elncRNAs (15 elncRNAs without eSNPs were removed) in each brain region, and extracted the eQTL beta coefficients, errors, and expression of the elncRNAs for subsequent analysis. Then, the Pearson correlation coefficient of each elncRNA expression level was calculated between two of the ten brain regions, and the correlation of errors in combination with the sample overlap was estimated based on the Bulik-Sullivan theory [21]. The variance of errors was approximated by the average of the square of the eQTL standard error. Using these statistics, we estimated the $r_{b}$ between the two brain regions by an extended Pearson correlation coefficient formula. Finally, the sampling variance of $r_{b}$ was calculated using the Jackknife approach, which excludes one elncRNA sequentially. See the original article for details on the algorithm [20].

**Genetic prediction model training and TWAS**

Firstly, we employed the PEER factors used for cis-eQTL analysis as covariates to conduct a multiple linear regression for each lncRNA and calculated the regression residuals to adjust the lncRNA expression matrices in each brain region. After integrating the adjusted lncRNA expression matrices, variant genotype data, and their corresponding annotation files (including IDs and locations of variants and lncRNAs), we trained the genetic prediction model in cis regions (within 1Mb of variant from lncRNA TSS) using the PredictDB pipeline that relies on an elastic net linear regression algorithm [22]. The trained model contains a database file and a SNP covariance matrices file. The database file undergoes further filtering based on the criterion of z-score P < 0.05 and average correlation coefficient (rho_avg) > 0.1. These two types of files were provided in lncBRAIN database. Finally, a large insomnia GWAS dataset comprising 1,331,010 individuals of European ancestry was utilized to perform TWAS analysis based on the trained model [23]. S-PrediXcan was employed to identify insomnia-related lncRNAs in each of the ten brain regions [24].

**mRNA quantification and 2SLS estimation**

Herein, we conducted a 2SLS estimation using eQTL data to avoid the impact of the potential unmeasured confounders while assessing the causal correlation between the exposure (lncRNA expression) and the outcome (mRNA expression). The LD-pruned variants significantly associated with lncRNA expression (eQTL nominal *P* < 10^-5^) are used as the instrumental variables. Firstly, the R package oligo with Affymetrix Exon 1.0 ST array annotation files was employed to quantify mRNAs from the same samples used for lncRNA quantification [25]. Then, each instrumental variable was subjected to eQTL analysis with all the 17,008 quantified mRNAs, and the variants with smaller nominal *P* values in mRNAs than in lncRNAs were filtered out to satisfy the exclusion criteria. Subsequently, three distinct metrics were applied for quality control: (1) weak instrument test and Sargan test were used to examine whether the instrumental variables were sufficiently strong to ensure the validity of their estimates in the first stage (*P* < 0.05); (2) Wu-Hausman test was applied to verify the absence of potential endogeneity in the instrumental variable regression model, ensuring the robustness of the analysis (*P* > 0.05). Finally, the 2SLS estimation of each lncRNA with all mRNA individually was conducted using R package ivreg (<https://CRAN.R-project.org/package=ivreg>), and the *P* values were adjusted by the Benjamini-Hochberg method to identify the significant protein-coding genes whose expression is influenced by each lncRNA (with a threshold of FDR *q* < 0.05).

**Database** **construction for lncRNA expression regulation in human brain**

The database lncBRAIN (<http://lncbrain.org.cn/>) visualizes the lncRNA expression, eQTL results, and the LD of the corresponding variants in the human brain. The results of lncRNA expression and eQTL analysis were visualized by box plots and distinguished according to gender, age, and brain region. The LD of variants in the cis-eQTL region was calculated and visualized using LDBlockShow software with its default parameters [26]. Users could easily query by lncRNA and SNP IDs, and download the results. The trained lncRNA prediction models and covariance matrices of the ten brain regions were also stored in lncBRAIN so users could to perform TWAS with their own data. We used HTML, CSS, and JavaScript to create interactive user interfaces, Python to handle back-end business logic and data flow, and MySQL to store the core data of the database.

**References**

1. Ramasamy A, Trabzuni D, Guelfi S. et al.. Genetic variability in the regulation of gene expression in ten regions of the human brain. *Nat Neurosci* 2014;17:1418-1428. 10.1038/nn.3801

2. Day IN. dbSNP in the detail and copy number complexities. *Hum Mutat* 2010;31:2-4. 10.1002/humu.21149

3. Gellert P, Ponomareva Y, Braun T. et al.. Noncoder: a web interface for exon array-based detection of long non-coding RNAs. *Nucleic Acids Res* 2013;41:e20. 10.1093/nar/gks877

4. Du Z, Fei T, Verhaak RG. et al.. Integrative genomic analyses reveal clinically relevant long noncoding RNAs in human cancer. *Nat Struct Mol Biol* 2013;20:908-913. 10.1038/nsmb.2591

5. Zheng Y, Xu Q, Liu M. et al.. lnCAR: A Comprehensive Resource for lncRNAs from Cancer Arrays. *Cancer Res* 2019;79:2076-2083. 10.1158/0008-5472.CAN-18-2169

6. Yates AD, Achuthan P, Akanni W. et al.. Ensembl 2020. *Nucleic Acids Res* 2020;48:D682-D688.

7. Jiang H, Wong WH. SeqMap: mapping massive amount of oligonucleotides to the genome. *Bioinformatics* 2008;24:2395-2396. 10.1093/bioinformatics/btn429

8. Carvalho B, Bengtsson H, Speed TP. et al.. Exploration, normalization, and genotype calls of high-density oligonucleotide SNP array data. *Biostatistics* 2007;8:485-499. 10.1093/biostatistics/kxl042

9. Shabalin AA. Matrix eQTL: ultra fast eQTL analysis via large matrix operations. *Bioinformatics* 2012;28:1353-1358. 10.1093/bioinformatics/bts163

10. Stegle O, Parts L, Piipari M. et al.. Using probabilistic estimation of expression residuals (PEER) to obtain increased power and interpretability of gene expression analyses. *Nat Protoc* 2012;7:500-507. 10.1038/nprot.2011.457

11. Consortium GT. The GTEx Consortium atlas of genetic regulatory effects across human tissues. *Science* 2020;369:1318-1330. 10.1126/science.aaz1776

12. Consortium GT. Human genomics. The Genotype-Tissue Expression (GTEx) pilot analysis: multitissue gene regulation in humans. *Science* 2015;348:648-660. 10.1126/science.1262110

13. Carlevaro-Fita J, Liu L, Zhou Y. et al.. LnCompare: gene set feature analysis for human long non-coding RNAs. *Nucleic Acids Res* 2019;47:W523-W529. 10.1093/nar/gkz410

14. Li X, Kim Y, Tsang EK. et al.. The impact of rare variation on gene expression across tissues. *Nature* 2017;550:239-243. 10.1038/nature24267

15. Sollis E, Mosaku A, Abid A. et al.. The NHGRI-EBI GWAS Catalog: knowledgebase and deposition resource. *Nucleic Acids Res* 2023;51:D977-D985. 10.1093/nar/gkac1010

16. Pezzella P. The ICD-11 is now officially in effect. *World Psychiatry* 2022;21:331-332. 10.1002/wps.20982

17. Yang H, Wang K. Genomic variant annotation and prioritization with ANNOVAR and wANNOVAR. *Nat Protoc* 2015;10:1556-1566. 10.1038/nprot.2015.105

18. Pruitt KD, Tatusova T, Maglott DR. NCBI reference sequences (RefSeq): a curated non-redundant sequence database of genomes, transcripts and proteins. *Nucleic Acids Res* 2007;35:D61-65. 10.1093/nar/gkl842

19. Hammal F, de Langen P, Bergon A. et al.. ReMap 2022: a database of Human, Mouse, Drosophila and Arabidopsis regulatory regions from an integrative analysis of DNA-binding sequencing experiments. *Nucleic Acids Res* 2022;50:D316-D325. 10.1093/nar/gkab996

20. Qi T, Wu Y, Zeng J. et al.. Identifying gene targets for brain-related traits using transcriptomic and methylomic data from blood. *Nat Commun* 2018;9:2282. 10.1038/s41467-018-04558-1

21. Bulik-Sullivan B, Finucane HK, Anttila V. et al.. An atlas of genetic correlations across human diseases and traits. *Nat Genet* 2015;47:1236-1241. 10.1038/ng.3406

22. Gamazon ER, Wheeler HE, Shah KP. et al.. A gene-based association method for mapping traits using reference transcriptome data. *Nat Genet* 2015;47:1091-1098. 10.1038/ng.3367

23. Jansen PR, Watanabe K, Stringer S. et al.. Genome-wide analysis of insomnia in 1,331,010 individuals identifies new risk loci and functional pathways. *Nat Genet* 2019;51:394-403. 10.1038/s41588-018-0333-3

24. Barbeira AN, Dickinson SP, Bonazzola R. et al.. Exploring the phenotypic consequences of tissue specific gene expression variation inferred from GWAS summary statistics. *Nat Commun* 2018;9:1825. 10.1038/s41467-018-03621-1

25. Carvalho BS, Irizarry RA. A framework for oligonucleotide microarray preprocessing. *Bioinformatics* 2010;26:2363-2367. 10.1093/bioinformatics/btq431

26. Dong SS, He WM, Ji JJ. et al.. LDBlockShow: a fast and convenient tool for visualizing linkage disequilibrium and haplotype blocks based on variant call format files. *Brief Bioinform* 2021;22. 10.1093/bib/bbaa227
